# Examining the Use of Consumer Wearable Devices and Digital Tools for Stress Measurement in College Students: Scoping Review of Methods

**DOI:** 10.2196/64144

**Published:** 2026-03-30

**Authors:** Aarti Sathyanarayana, Ohida Binte Amin, Jennie An, Jukka Pekka Onnela

**Affiliations:** 1 Bouve College of Health Sciences, Northeastern University, 360 Huntington Avenue, Boston, MA, 02115, United States; 2 Khoury College of Computer Science, Northeastern University, Boston, MA, United States; 3 Department of Biostatistics, Harvard T.H. Chan School of Public Health, Harvard University, Boston, MA, United States

**Keywords:** digital phenotyping, wearable technology, stress detection, machine learning, college students

## Abstract

**Background:**

College-aged students face persistent academic and social stress that adversely affects their mental and physical health. Digital phenotyping with wearable devices enables real-time stress monitoring from continuous physiological signals, supporting just-in-time therapeutic interventions to improve student well-being. Despite rapid advances in wearables and analytical methods, it remains unclear which devices, physiological signals, and machine learning or deep learning approaches are most commonly used for stress detection in this population.

**Objective:**

This study aimed to systematically review the literature to identify best practices and emerging trends in stress measurement using wearable technology and digital tools among college-aged students. We sought to evaluate commonalities in sensor types, datasets, and machine learning approaches used for stress detection.

**Methods:**

A systematic search was conducted across medical and computer science databases, including Embase, PubMed, IEEE Xplore, and ACM Digital Library, for studies published between January 2020 and December 2025. Studies were included if they examined psychological stress detection using wearable or digital tools among college-aged students and were excluded if they focused on nonpsychological stress, were reviews or prototypes without a defined study population, or lacked clear population information. Two reviewers independently screened studies and extracted data on the wearable sensors, physiological signals, datasets, and modeling approaches to summarize trends in stress prediction.

**Results:**

A total of 134 studies met the inclusion criteria and were included in the review from the original 792 papers. Electrodermal activity was the most frequently used physiological signal, appearing in 57.5% (n=77) of studies, and wrist-worn wearable devices were the predominant sensing modality. Among studies that compared algorithms, support vector machines were identified as the most commonly applied and best-performing model in 33.3% (n=45) of cases. Overall, 62.8% (n=84) of included studies relied on preexisting datasets, and approximately 80% (n=67) of those used the Wearable Stress and Affect Detection dataset, which contains only 15 participants. Demographic reporting was inconsistent, as 27.6% (n=37) of studies did not report sex distribution, and only 4 studies justified the sample size. The use of temporal modeling algorithms was limited, despite their importance for capturing the dynamic, time-varying nature of stress. This review highlights persistent gaps and underscores the need for more diverse datasets and advanced modeling approaches to improve stress detection accuracy.

**Conclusions:**

Our review innovatively synthesizes wearable-based stress detection research focused on college-aged students. Unlike prior reviews that aggregate heterogeneous populations or focus primarily on algorithmic performance, this review focused on wearable sensors, physiological signals, modeling approaches, and methodological quality to identify persistent gaps limiting real-world deployment. These findings inform the development of more generalizable monitoring systems to support early mental health intervention in students.

## Introduction

With the widespread adoption of wearable devices, numerous stress monitoring frameworks have been designed specifically for undergraduate students [1-3], given their heightened susceptibility to psychological stress. This need is underscored by findings that over 80% of undergraduate students report experiencing significant stress related to their academic life [4]. University life can be particularly overwhelming, as many students experience independent living for the first time while navigating self-care and decision-making [5]. While positive stress can sometimes enhance academic performance, persistent and long-lasting chronic stress can negatively impact both mental and physical health [6]. By proactively managing stress, individuals can mitigate the risk of stress-related health issues, including cardiovascular problems, gastrointestinal issues, mental health disorders, substance abuse, and chronic diseases such as diabetes or hypertension [7]. Stress also significantly disrupts sleep [8], social interactions [9], and academic performance [10], contributing to insomnia [11], anxiety [12], and a weakened immune system [13]. Digital phenotyping of stress, leveraging wearable and mobile technologies, enables just-in-time stress management solutions that help prevent chronic stress from compromising long-term health.

In recent years, the use of consumer wearables to monitor physical activity [14] and other lifestyle traits [15] has become more prevalent. For example, many commercial consumer wearables are being used to keep track of and improve upon fitness regimens [16]. With this increased availability of wearables comes the possibility for real-time health management using these commercial devices that are more convenient and lightweight [17]. The use of wearables to passively monitor physiological signals and the subsequent analysis using various machine learning and deep learning models brings enormous benefits for health management [18]. By passively tracking heart rate (HR) or heart rate variability (HRV), skin temperature, electrodermal activity (EDA), electroencephalogram, electrocardiogram (ECG), acceleration, and other physiological variables, smartphones and wearable sensors can provide features related to signs indicative of poor mental health [19]. Stress is reflected in the body with increased EDA or HR, reflecting the autonomic nervous system and hypothalamic-pituitary-adrenal axis activity [20]. Many studies have tracked these biosignals with commercial digital tools to build models to measure stress [21]. In this review, we examine the trends in the current use of these digital tools to measure stress.

Stress assessment using wearable and digital technologies has been conducted across both controlled laboratory experiments and real-world, free-living conditions. In laboratory settings, studies commonly use well-established stress elicitation tasks [22] with resting periods used as baselines. Commonly used tasks include the Trier Social Stress Test (TSST), mental arithmetic tasks [23] (eg, the Montreal Imaging Stress Task [24]), the Stroop color-word test, public speaking, startle response tests, cold pressor tests, and stress-inducing video stimuli [25]. Across these studies, researchers used varying combinations of physiological signals and derived diverse feature sets following preprocessing steps such as artifact removal, signal normalization, and feature selection [26]. In contrast, stress monitoring in free-living environments relies on self-reported stress measures alongside passive and unobtrusive sensing approaches that capture daily physiological and behavioral patterns using wearable devices and smartphones [27]. These approaches vary widely in sensor availability, feature extraction methods, and contextual information, leading to substantial heterogeneity in how stress is represented and quantified across wearables and digital tools.

Alongside variability in study design, stress capture methods, and physiological sensing, approaches for stress prediction differ markedly across studies. Both traditional machine learning [28,29] and deep learning [30,31] models have been applied to physiological time-series data to identify stress episodes and enable just-in-time interventions. However, it remains unclear which modeling paradigms are most appropriate for different physiological signals and smartphone-derived active and passive sensing data, how model architectures should be designed to capture temporal stress dynamics, and whether increased model complexity consistently yields performance gains. These methodological challenges hinder the translation of wearable-based stress detection systems into practical tools for continuous monitoring and personalized support in college-aged populations, underscoring the need for systematic evidence synthesis and clearer methodological pathways for future research.

This review aims to identify trends in current research and highlight areas for improvement that future researchers should focus on. There is a need to understand which algorithms perform best, which wearables are most used, and which signals are most informative. The topic of this review is identifying moments of high stress using digital tools and ubiquitous data in college-aged students. We examine both machine learning and deep learning advancements in the field, as well as comparisons of methods, where a scoping review is the most appropriate synthesis method to address the stated objectives. Our population of interest includes college students aged 18‐24 years. Publication dates of interest include conference and journal papers published between 2020 and 2025, as we focus on advancements in the field, including newer wearable devices and algorithms. We are also narrowing our focus to college students, as university is a particularly stressful place where their health and lifestyle habits are likely to fluctuate [32]. Academic stress is directly linked to health crises such as anxiety and depression, indicating an opportunity to monitor stress and prevent health from deteriorating [33]. In this scoping review, we summarize the wearables used, signals measured, and algorithms performed to measure stress. We then discuss trends in data and practices across papers. We conduct a quality assessment of all included studies. We also provide an overview of the results and a discussion of limitations and future possibilities for stress measurement. As a result, this scoping review aims to synthesize recent research on wearable or digital tool–based stress detection among college-aged students by summarizing the sensing technologies used, the physiological and behavioral signals measured, the machine learning and deep learning models applied, and key methodological practices, to identify current trends, limitations, and directions for future research.

## Methods

### Overview

We conducted a scoping review to characterize current research on stress detection using wearable and digital tools among college-aged students. This review synthesizes studies published between January 2020 and December 2025 to summarize commonly used wearable devices, physiological signals, datasets, and machine learning or deep learning approaches for identifying high-stress moments. By organizing existing methods and conducting a quality assessment, this review provides an overview of methodological practices and highlights areas for future research in wearable-based stress measurement. This scoping review adhered to the methodological framework proposed by Arksey and O’Malley [34], which includes identifying the research question, identifying relevant studies, study selection, charting the data, and collating, summarizing, and reporting the results. Finally, this scoping review was conducted and reported in accordance with the PRISMA-ScR (Preferred Reporting Items for Systematic Reviews and Meta-Analyses extension for Scoping Reviews) guidelines to ensure transparency and reproducibility [35].

### Protocol and Registration

No formal review protocol was registered for this scoping review, as the objective was to map the scope and characteristics of existing evidence in stress prediction research using wearable technology.

### Eligibility Criteria

We defined eligibility criteria to ensure that only relevant and methodologically appropriate studies were included in this review. Studies were included if they measured or classified psychological stress using physiological signals from a tool, wearable, or sensor. Only experimental or observational studies published in English were considered. The target population was college students aged 18‐24 years. Studies that partially included this age range were eligible if they explicitly mentioned students as a distinct group or if the mean age, along with the SD, fell within the target population. Studies were excluded if they focused on nonpsychological stress (eg, mechanical stress), were review papers, extended abstracts, or prototype descriptions without a defined study population. Papers without clear population details or those identifying participants solely by employment (eg, “office workers” or “hospitalized patients”) were also excluded.

### Information Sources

We searched IEEE Xplore, ACM Digital Library, PubMed, and Embase for conference and journal papers covering studies published between January 2020 and December 2025, a time frame selected to capture recent developments in wearable sensing technologies and stress detection methodologies.

### Search

We used a combination of terms related to the key concepts of psychological stress, wearable devices, and sensors (full search per database is provided in Multimedia Appendix 1). We extracted each database searched and the platform used, including IEEE Xplore, ACM Digital Library, PubMed, and Embase, in accordance with PRISMA-S (PRISMA literature search extension) [36], and all databases were searched independently rather than through a multidatabase platform. No multidatabase searching or study registry searching was conducted. No additional online resources (eg, tables of contents, print conference proceedings, and websites) were browsed. No additional search methods were used, including citation searching, contacting authors or experts, or setting up citation alerts. The full search strategies for each database are provided in Multimedia Appendix 1, including the specification that no filters or limits other than language (English) and publication date (January 2020 to December 2025) were applied. Search strategies were developed with input from 2 academic librarians; however, search strategies from prior reviews were not reused, and no formal peer review of the search strategy was conducted. No additional methods were used to update the search. Therefore, searches were limited to studies published in English within the specified date range. No restrictions were applied based on study design. All retrieved records were initially screened. Following screening, records were imported into Rayyan (Rayyan Systems Inc) [37], where duplicate entries were identified and removed. The deduplicated set of records was then used for abstract and full-text screening.

### Selection of Sources of Evidence

Two independent reviewers screened all records using a 2-stage selection process. Studies were checked for eligibility by 2 reviewers independently screening titles and abstracts. This first round of filtering focused on relevance. Abstracts were also screened for population. Some papers did not mention population in the abstract and were thus moved to full-text screening. This resulted in 261 papers for full-text screening. During this second round of filtering, studies were also checked for eligibility by 2 researchers independently reviewing the full text. Disagreements at any stage of eligibility and filtering were resolved by the 2 reviewers discussing their reasons for either inclusion, exclusion, or neither. Full agreement was reached for abstract and full-text screening, leading to the final inclusion of 134 papers.

### Data Charting

A standardized data-charting form was jointly developed by 2 reviewers to identify and extract relevant information aligned with the review objectives. The form was pilot-tested on a subset of included studies and refined iteratively to ensure completeness and consistency. Two reviewers independently charted data from all eligible studies, compared their entries, and resolved discrepancies through discussion. All data were extracted directly from the published papers, and no additional information was sought from study authors.

### Data Items

To extract consistent information from each paper, we conducted systematic data extraction as outlined in Tables 1-3. Extracted variables included study details (title, authors, publication date, study purpose, and data collection duration), sample characteristics (age, sex, sample size, and demographic information), sensor type, and all available feature categories used in the study (sleep, physiological signals, calorie intake or expenditure, phone use, activity, location, and survey or EMA data). For studies conducting algorithm comparisons, we additionally extracted the types of signals analyzed, devices used, algorithms tested, performance measures, best-performing algorithm, validation strategy, and outcome measures.

**Table 1.** Summary characteristics of 134 included studies.

Study	Sample (n)	Sex	Age (years), mean (SD)	Sleep	Physiological signals	Calorie intake or expenditure	Phone use	Activity	Location	Survey	Total feature types
Bellante et al [38]	15	3 females and 12 males	27.5 (2.4)		✔						1
Faro and Giordano [39]	—^a^	—	College students		✔			✔	✔		3
Faro et al [40]	31	—	College students		✔						1
Iranfar et al [41]	95	95 males	20.43 (2.17)		✔						1
Mohammadi et al [42]	18	5 females and 13 males	27.5 (2.4)		✔						1
Mustafa et al [43]	15	3 females and 12 males	27.5 (2.4)		✔						1
Arsalan and Majid [44]	40	20 females and 20 males	24.86 (6.69)		✔						1
Li and Sano [45]	239	—	College students		✔			✔			2
Can et al [27]	14	5 females and 9 males	23.5 (N/A)^a^		✔						1
Cheadle et al [46]	100	61 females and 39 males	20.4 (N/A)		✔						1
Chen et al [47]	30	20 females and 10 males	23 (NA)							✔	1
Gupta et al [48]	15	3 females and 12 males	27.5 (2.4)		✔			✔			2
Panganiban and de Leon [49]	36	—	21.5 (N/A)		✔						1
Gasparini et al [50]	36	14 females and 22 males	24.7 (3.3)		✔						1
Azgomi et al [51]	20	—	College students		✔			✔			2
Yu and Sano [31]	243	—	College students		✔			✔			2
Han et al [52]	17	4 females and 13 males	24 (N/A)		✔						1
Wu et al [53]	264	113 females and 151 males	22.8 (N/A)		✔			✔			2
Jelsma et al [54]	100	—	College students		✔						1
Lai et al [55]	15	3 females and 12 males	27.5 (2.4)		✔						1
Liakopoulos et al [56]	Multiple datasets	Multiple datasets	Multiple datasets		✔						1
Li and Sano [57]	239	—	College students		✔			✔			2
Hssayeni and Ghoraani [58]	15	3 females and 12 males	27.5 (2.4)		✔			✔			2
Gil-Martin et al [59]	15	3 females and 12 males	27.5 (2.4)		✔			✔			2
Han et al [60]	20	—	College students		✔						1
Mishra et al [61]	27	15 females and 12 males	23 (3.24)		✔			✔		✔	3
Mishra et al [26]	90	—	Graduate and undergraduate students		✔						1
Momeni et al [62]	60	60 males	20.43 (2.17)		✔						1
Rashid et al [63]	15	3 females and 12 males	27.5 (2.4)		✔						1
Bobade and Vani [18]	15	3 females and 12 males	27.5 (2.4)		✔			✔			2
Yannam et al [64]	70	—	Undergraduate	✔	✔		✔	✔	✔		5
Pakhomov et al [65]	18	14 females and 4 males	20.1 (2.01)		✔			✔			2
Holder et al [66]	11	10 females and 1 male	27.5 (2.4)		✔			✔			2
Elzeiny and Qaraqe [67]	22	5 females and 17 males	27.5 (2.4)		✔						1
Heo et al [68]	15	3 females and 12 males	27.5 (2.4)		✔						1
Kar et al [69]	15	3 females and 12 males	27.5 (2.4)		✔			✔			2
Prashant et al [70]	15	3 females and 12 males	27.5 ( 2.4 )		✔						1
Samyoun et al [71]	15	3 females and 12 males	27.5 ( 2.4 )		✔						1
Silva et al [72]	82	63 females and 19 males	22.13 (5.55)	✔	✔	✔					3
Islam et al [73]	20	7 females, 12 males, and 1 nonbinary	22 (N/A)	✔	✔			✔	✔		4
Vidal et al [32]	49	25 females and 24 males	18.1 (N/A)	✔						✔	2
Wu et al [74]	169	81 females and 88 males	22.8 (6.2)		✔						1
Mitro et al [75]	30	22 males and 8 females	27.5 (2.4)		✔						1
Zhu et al [28]	112	—	—		✔						3
Tutunji et al [76]	84	32 males and 52 females	College students		✔					✔	5
Lange et al [77]	15	12 males	27.5 (2.4)		✔						4
Abdul et al [78]	20	—	—		✔						2
Almadhor et al [79]	15	12 males and 3 females	27.5 (2.4)		✔						6
Vos e al [29]	136	—	—		✔						13
Mai and Chung [80]	15	—	30 (7)		✔					✔	2
Sepanloo et al [81]	12	—	29.6 (10.1)		✔						3
Gedam et al [2]	200	128 male and 72 female	23 (N/A)		✔						3
Darwish et al [82]	1017	496 males and 454 females	27.5 (2.4)		✔						3
Lim et al [83]	5	4 males and 1 female	—		✔						2
Bloomfield et al [3]	525	144 males and 381 females	22 (N/A)	✔	✔					✔	6
Nazeer et al [84]	15	12 males and 3 females	27.5 (2.4)		✔						6
Almadhor et al [85]	15	12 males and 3 females	27.5 (2.4)		✔						6
Stržinar et al [86]	15	12 males and 3 females	27.5 (2.4)		✔						1
Chen and Lee [30]	30	6 males and 24 females	20.4 (N/A)		✔						3
Feng et al [87]	15	12 males and 3 females	27.5 (2.4)		✔						6
Xuanzhi et al [88]	15+	—	—		✔						2
Vidal et al [89]	55	—	18.5 (N/A)	✔	✔					✔	2
Fauzi et al [90]	15	12 males and 3 females	27.5 (2.4)		✔						4
Tazarv et al [91]	20	13 males and 7 females	25 (N/A)		✔						4
Alfredo et al [92]	35	—	—		✔						4
Su et al [93]	18403	8565 males and 9838 females	118.5 (N/A)							✔	1
Wang et al [94]	15	12 males and 3 females	27.5 (2.4)		✔						1
Can and André [95]	14	9 males and 5 females	23 (N/A)		✔						3
Prajod et al [96]	135	—	—		✔						4
Ganesan et al [97]	15	12 males and 3 females	27.5 (2.4)		✔						7
Sun et al [98]	21	—	23 (2.91)		✔					✔	2
Neigel et al [99]	103	91 males and 12 females	21.8 (1.9)	✔	✔			✔			4
Pogliaghi et al [100]	15	12 males and 3 females	27.5 (2.4)		✔						2
Jaiswal et al [101]	64	—	—		✔						1
Rashid et al [102]	15	12 males and 3 females	27.5 (2.4)		✔						7
Narwat et al [103]	15	12 males and 3 females	27.5 (2.4)		✔						3
Kafková et al [104]	15+	—	—		✔						2
Lopez et al [105]	166	—	21 (N/A)	✔	✔	✔		✔			5
Wilfred et al [106]	25	—	—		✔						2
Jaiswal et al [107]	60	—	—		✔						1
Gaitan-Padilla et al [108]	12	5 males and 7 females	—		✔						2
Gupta et al [109]	15	12 males and 3 females	27.5 (2.4)		✔						3
Beierle and Pryss [110]	15	12 males and 3 females	27.5 (2.4)		✔						4
Masrur et al [111]	15+	—	College students		✔						1
Sakanti et al [112]	15	12 males and 3 females	27.5 (2.4)		✔						6
Shedage et al [113]	15	12 males and 3 females	27.5 (2.4)		✔						7
Gaitan-Padilla et al [114]	5	4 males and 1 female	22.6 (0.55)		✔						2
Tanwar et al [115]	15	12 males and 3 females	27.5 (2.4)		✔						6
Gullapalli et al [116]	18	—	20 (N/A)		✔						1
Sadruddin et al [117]	15	12 males and 3 females	27.5 (2.4)		✔						6
Jahanjoo et al [118]	15	12 males and 3 females	27.5 (2.4)		✔						1
Parousidou et al [119]	15	12 males and 3 females	27.5 (2.4)		✔						6
Karpagam et al [120]	15	12 males and 3 females	27.5 (2.4)		✔						3
Sethia et al [121]	36	32 males and 4 females	21 (N/A)		✔						4
Hasanpoor et al [122]	15	12 males and 3 females	27.5 (2.4)		✔						1
Benita et al [123]	15	12 males and 3 females	27.5 (2.4)		✔						1
Hsu [124]	10	—	College students		✔						1
Carmisciano et al [125]	15	12 males and 3 females	27.5 (2.4)		✔						2
Warrier et al [126]	15	12 males and 3 females	27.5 (2.4)		✔						5
Calbert and Tonekaboni [127]	5	2 males and 3 females	College students		✔						4
Hoang et al [1]	15	12 males and 3 females	27.5 (2.4)		✔						6
Kumar et al [128]	15	12 males and 3 females	27.5 (2.4)		✔						6
Hasanpoor et al [129]	15	12 males and 3 females	27.5 (2.4)		✔						1
Le et al [130]	10	—	College students		✔						3
Fernandez et al [131]	30	15 males and 15 females	28 (N/A)		✔						1
Tanwar et al [132]	15	12 males and 3 females	27.5 (2.4)		✔						3
Huang et al [133]	15	12 males and 3 females	27.5 (2.4)		✔						1
Oh et al [134]	15	12 males and 3 females	27.5 (2.4)		✔						6
Thapa et al [135]	15	12 males and 3 females	27.5 (2.4)		✔						6
Abdelfattah et al [136]	15	12 males and 3 females	27.5 (2.4)		✔						6
Tsiampa et al [137]		—	College students		✔						1
Fazeli et al [138]	14	—	College students		✔	✔		✔			8
Subathra and Malarvizhi [139]	15	12 males and 3 females	27.5 (2.4)		✔						2
Shikha et al [140]	36	—	20 (N/A)		✔						3
Andreas et al [141]	15	12 males and 3 females	27.5 (2.4)		✔						6
Lee et al [21]	15	12 males and 3 females	27.5 (2.4)		✔						6
Kasnesis et al [142]	15	12 males and 3 females	27.5 (2.4)		✔						6
Ciharova et al [143]	42	13 males and 29 females	20.79 (N/A)		✔						2
Darwish et al [144]	15	12 males and 3 females	27.5 (2.4)		✔						3
Nuamah [145]	32	—	25.2 (2.3)		✔						2
Saylam and İncel [19]	700	—	College students	✔	✔			✔			4
Sa-nguannarm et al [146]	15	12 males and 3 females	27.5 (2.4)		✔						6
Nelson et al [147]	103	—	College students		✔		✔			✔	3
Dahal et al [148]	15	12 males and 3 females	27.5 (2.4)		✔						1
Aqajari et al [149]	11	4 males and 7 females	22.91 (5.05)		✔						1
Jiao et al [150]	32	14 males and 18 females	22.69 (3.73)		✔						1
Yuting and Rashid [33]	502	476 males and 26 females	College students	✔				✔		✔	3
Lotfi et al [151]	168	168 females	122.5 (N/A)					✔			3
Belwafi et al [23]	36	8 males and 28 females	21 (N/A)		✔						1
Patanè et al [152]	16	—	College students				✔	✔			3
Subathra et al [153]	46	40 males and 6 females	22 (N/A)		✔						2
Li et al [25]	177	89 males and 88 females	20.37 (2.97)		✔					✔	3
Van der Mee et al [154]	95	15 males and 80 females	20 (N/A)		✔					✔	2
Rosenbach et al [24]	60	20 males and 40 females	27.5 (5.6)		✔						3

a Not available.

**Table 2.** Details for studies conducting algorithm comparisons.

Study	Device used	Physiological or nonphysiological signals	Algorithm	Performance measure	Best performing algorithm	Validation
Bellante et al [38]	Wrist and chest devices	BVP^a^, EDA^b^, and ESP^c^	DT^d^, bagging DT, RF^e^, Extra Trees, AdaBoost^f^ DT, SVM^g^, KNN^h^, LR^i^, and LDA^j^	Accuracy and *F*_1_-score	SVM	Leave-one-out cross-validation (LOOCV)
Iranfar et al [41]	Biopac BioNomadix System	EDA, RESP^k^, ECG^l^, and PPG^m^	LDA, SVM, RF, XGBoost^n^, Isolation forest, and Bayesian ridge algorithm	Accuracy	XGBoost	Group k-fold cross-validation (k=10)
Mohammadi et al [42]	—^o^	ECG and EDA	KNN, DT, RF, SVM, and FCM^p^	Accuracy, sensitivity, and specificity	KNN	Train and test split
Mustafa et al [43]	SA9309M, AD8232, and MAX30205	HR^q^, SC^r^, and TEMP^s^	ANN^t^, KNN, DT, and SVM	Accuracy	DT	Train and test split
Arsalan and Majid [44]	MUSE EEG^u^, Shimmer GSR^v^, and PPG optical pulse clip	EEG, GSR, and PPG	KNN, DT, RF, MLP^w^, and SVM	Accuracy and *F*_1_-score	SVM	LOOCV
Can et al [27]	Smartwatch and Empatica E4	EDA and HR	MLP, RF (n=100), KNN (n=3), SVM, and LR	Accuracy	RF and SVM	10-fold CV^x^
Panganiban and de Leon [49]	Smartphone and CorSense	PRV^y^ from PPG	KNN, NN^z^, SVM, RF, and AdaBoost	Accuracy	RF	Stratified k-fold CV
Gasparini et al [50]	Shimmer3 GSR	BVP	SVM linear kernel and CNN^aa^	Accuracy, precision, recall, and *F*_1_-score	CNN	Train and test split
Yu and Sano [31]	Wrist device and Android phone data	ACC^ab^, SC, and TEMP	LSTM^ac^, combination of LSTM and CNN	MAE^ad^ and statistical analyses	LSTM	5-fold CV
Han et al [52]	Shimmer3 ECG, Shimmer 3 GSR+, and Empatica E4	ECG, PPG, and GSR	KNN (k=1, 3, 5, 7, and 9), SVM, and Naïve Bayes classifier	Accuracy	SVM	10-fold CV
Liakopoulos et al [56]	Body sensors, wrist, and chest devices	ECG, EDA, and HR	CNN, SVM, KNN, RF, and NN	Accuracy and *F*_1_-score	SVM	10-fold and LOSO^af^ CV
Hssayeni and Ghoraani [58]	Wrist and chest devices	RESP, ECG, EMA^ag^, EDA, TEMP, and ACC	Gradient tree boosting and CNN	MAE and r	CNN	LOOCV
Mishra et al [61]	Polar H7, Amulet wrist, and custom-made GSR sensor	HR, activity data, EMA prompts, and GSR	SVM and RF	Accuracy and *F*_1_-score	SVM	LOOCV
Mishra et al [26]	Polar H10, Polar H7, and Empatica E4	HR and EDA	SVM and RF	Precision, recall, and *F*_1_-score	SVM with HR, RF for HR and EDA	LOOCV
Bobade and Vani [18]	Wrist and chest devices	ACC, ECG, BVP, TEMP, RESP, EMG^ah^, and EDA	KNN, LDA, RF, DT, AdaBoost, Kernel SVM, and ANN	Accuracy	ANN	LOOCV
Elzeiny and Qaraqe [67]	PPG sensor and Empatica E4	IBI^ai^ and BVP	CNN, RF, Extra Trees, extremely randomized trees, and SVM	Accuracy	CNN and Extra Trees	CNN: 5-fold cross validation and ML:^aj^ 10-fold cross validation
Prashant et al [70]	Wrist and chest devices	ECG	LDA, RF (100 base estimators), SVM (Gaussian kernel), and ANN	Accuracy	RF	Train and test split
Silva et al [72]	Microsoft Smartband 2	HR, SC, TEMP, calorie intake and expenditure, and sleep patterns	Logistic regression, NN, Naïve Bayes, SVM, RF, and KNN	Sensitivity and specificity	NN	Train and test split
Islam et al [73]	Fitbit Charge 2 and Android	HR, sleep, step count, GPS location, sound intensity, and light data	LR, KNN, SVM, and NN	Accuracy	SVM	10-fold CV
Zhu et al [28]	Empatica E4, Affectiva Q Curve, and Shimmer3	EDA, PPG, and ECG	SVM, RF, KNN, Naïve Bayes, and LR	Accuracy, recall, precision, and *F*_1_-score	SVM	LOSO and 10-fold CV
Sepanloo et al [81]	Empatica E4 and Zephyr BioHarness 3 chest straps	HR, EDA, and TEMP	RF, gradient boosting classifier, and stacking models	Accuracy, precision, recall, *F*_1_-score, and support	Stacking models	Stratified 5-fold CV
Gedam et al [2]	Empatica E4 and RespiBAN	ECG, GSR, and TEMP	KNN, SVM, DT, RF, AdaBoost, XGBoost^n^, and gradient boosting	Accuracy, precision, recall, *F*_1_-score, and AUC^ak^	XGBoost	Train and test split and 10-fold CV
Alfredo et al [92]	Empatica E4	TEMP, EDA, BVP, and salivary cortisol	SVM, AdaBoost, RF, LDA, and KNN	Accuracy	RF and KNN	Train and test split
Su et al [93]	—	Self-reports (PSQI^al^, DASS-21^am^, CD-RISC^an^, and IPAQ)^ao^	RF LR, SVM, and FNN^ap^	Accuracy, specificity, and *F*_1_-score	RF	Train and test split
Wang et al [94]	Empatica E4 and RespiBAN	HRV^ae^	SVM and KNN	Accuracy, *F*_1_-score, recall, and precision	SVM	10-fold CV
Prajod et al [96]	RespiBAN, Empatica E4, TMSI Mobi, IOM biofeedback device, and Actiwave Cardio Monitor	ECG, EDA, BVP, and TEMP	RF, SVM, and MLP	*F*_1_-score and accuracy	RF	LOSO
Narwat et al [103]	RespiBAN	EDA, ECG, and TEMP	CNN, KNN, and XGBoost	Accuracy, precision, recall, *F*_1_-score, and support	CNN	—
Sadruddin et al [117]	Empatica E4 and RespiBAN	ECG, EDA, EMG, ACC, TEMP, and RESP	DT, XGBoost, LR, and LDA	Accuracy	XGBoost	10-fold CV
Jahanjoo et al [118]	Empatica E4 and RespiBAN	PPG	KNN, LDA, SVM, DT, RF, and AdaBoost	Accuracy	SVM	CV
Karpagam et al [120]	Empatica E4	ACC, EDA, and TEMP	RF and LR	Accuracy	RF	10-fold CV
Hsu [124]	Empatica E4	EDA	LDA, SVM, and KNN	Precision, recall, *F*_1_-score, and accuracy	SVM	Train and test split
Calbert and Tonekaboni [127]	Hexoskin vests and Actigraph watches	HR, RESP, breathing volume, and movement	RF, KNN, XGBoost, and NN	Accuracy	RF	LOSO
Le et al [130]	Empatica E4	HR, EDA, and TEMP	SVM and KNN	*F*_1_-score and accuracy	KNN	10-fold CV
Fernandez et al [131]	EEG Enobio device and the BIOPAC MP36	EEG	LightGBM^aq^, CNN, KNN, and SVM	Accuracy	LightGBM	Train and test split and 5-fold CV
Shikha et al [140]	Empatica E4	EDA, PPG, and ACC	Gradient Boosting, SVM, KNN, RF, and EBM^ar^	Accuracy	Gradient boosting	—
Aqajari et al [149]	Samsung Galaxy Gear Sport watches	PPG	KNN, RF, and XGBoost	*F*_1_-score	RF	5-fold CV

a BVP: blood volume pulse.

b EDA: electrodermal activity.

c ESP: echo squeezing protocol.

d DT: decision tree.

e RF: random forest.

f AdaBoost: adaptive boosting.

g SVM: support vector machine.

h KNN: k-nearest neighbor.

i LR: logistic regression.

j LDA: linear discriminant analysis.

k RESP: response.

l ECG: electrocardiogram.

m PPG: photoplethysmography.

n XGBoost: extreme gradient boosting.

o Not available.

p FCM: fuzzy c-means.

q HR: heart rate.

r SC: skin conductance.

s TEMP: temperature.

t ANN: artificial neural network.

u EEG: electroencephalogram.

v GSR: galvanic skin response.

w MLP: multilayer perceptron.

x CV: cross-validation.

y PRV: pulse rate variability.

z NN: neural network.

aa CNN: convolutional neural network.

ab ACC: accelerometer.

ac LSTM: long short-term memory.

ad MAE: mean absolute error.

ae HRV: heart rate variability.

af LOSO: leave-one-subject-out.

ag EMA: ecological momentary assessment.

ah EMG: electromyography.

ai IBI: interbeat interval.

aj ML: machine learning

ak AUC: area under the receiver operating characteristic curve.

al PSQI: Pittsburgh Sleep Quality Index.

am DASS-21: Depression Anxiety Stress Scales–21.

an CD-RISC: Connor–Davidson Resilience Scale.

ao IPAQ: International Physical Activity Questionnaire.

ap FNN: feedforward neural network.

aq LightGBM: light gradient boosting machine.

ar EBM: explainable boosting machine.

**Table 3.** Details for studies testing or comparing their own framework or conducting statistical analyses.

Study	Device used	Features used	Algorithm analysis	Performance measure	Results	Validation
Faro and Giordano [39]	ECG^a^ wearable and wearable body sensor network	HR^b^, activity, time, and location	ANN^c^ and SOM^d^ for proposed framework	Classification tool	Model successful	Train/test split
Faro et al [40]	ECG wearable and wearable body sensor network	HR	SOFM^e^	—^f^	Defined as accurate enough	Train/test split
Li and Sano [45]	Wrist	SC^g^, TEMP^h^, and ACC^i^	L2 and 1-norm regularized multitask least squares regression	Mean squared error and MAE^j^	Early fusion better	Train/test split
Cheadle et al [46]	SAM^k^ activity wearable, EDA^l^ sensor, and Empatica E4	EDA^l^	Linear regression	Statistical correlation	Support prior findings that perceived microaggressive discrimination increases negative emotion	—
Chen et al [47]	Personalized system and surveys	Survey questions	Proposed framework	MAE	—	—
Gupta et al [48]	RespiBAN and Empatica E4	ECG, EMG^m^, TEMP, RESP^n^, BVP^o^, EDA, and ACC	CNN^p^ and k-medoid clustering	Accuracy and execution time	Success	4-fold CV^q^
Azgomi et al [51]	Affectiva Q Curve and Nonin Wireless WristOx2 oximeter	SC, TEMP, ACC, HR, and blood oxygenation	Bayesian filtering with an expectation maximization (EM)	*t* test comparison	Success	—
Wu et al [53]	Wrist and smartphone	EDA, PPG^r^, TEMP, and ACC	Proposed framework and SVM^s^	Accuracy	Framework proposed	5-fold CV
Jelsma et al [54]	Wrist-worn EDA sensor, Empatica E4, and smartphone	EDA	Econometric fixed-effects with robust SE regression approach	Statistical analyses	—	—
Lai et al [55]	Wearable body sensor network	TEMP and EDA	Proposed framework with Res-TCN^t^ classifier	Accuracy	High accuracy	LOOCV^u^
Li and Sano [57]	Wrist	TEMP, SC, and ACC	MTL^v^ linear regression model and k-means clustering for the proposed framework	MSE^w^ and MAE	The framework can extract features better than feature crafting or static autoencoders, and temporal features demonstrated significantly higher precision than static and crafted features.	4-fold CV
Gil-Martin et al [59]	RespiBAN and Empatica E4	ACC, TEMP, RESP, ECG, EMG, EDA, and BVP	CNN	Accuracy and *F*_1_	—	LOOCV
Han et al [60]	Wrist	EDA, TEMP, ACC, HR, and blood oxygenation	Adversarial networks and transfer learning	Accuracy	Disentangled adversarial transfer learning framework	LOOCV
Momeni et al [62]	Biopac system	ECG, RESP, PPG, and EDA	XGBoost^x^ algorithm	Accuracy and *F*_1_	—	Group Shuffle Split CV with 10 iterations.
Rashid et al [63]	Wrist-based PPG sensor	BVP	CNN	Accuracy and *F*_1_	Success	LOOCV
Yannam et al [64]	Smartphones (Android) and fitness trackers (eg, OnePlus Band)	User screen time, devices around user, mobile and application usage stats, mobile interaction, location data, HR, sleep data, and step counts	Proposed framework	—	—	—
Pakhomov et al [65]	Fitbit	HR and activity	*t* test, significance levels, and Spearman rank test	—	—	—
Holder et al [66]	Empatica E4	ACC, BVP, EDA, and TEMP	KNN^y^, DT^z^, and CNN	Accuracy and *F*_1_	Single modality showed promise	LOOCV
Heo et al [68]	PPG sensor	HR	DT, RF^aa^, Ada-boosting^ab^, 9-NN^ac^, LDA^ad^, SVM, gradient-boosting, and the proposed framework OMDP^ae^	Accuracy and *F*_1_	OMDP	LOOCV
Kar et al [69]	Wrist and chest	ACC, EDA, and TEMP	Binary classifier based on GRU^af^ and RNN^ag^	Precision, recall, *F*_1_, and accuracy	Support the use of a modest set of signals that are easily collected on wearables.	
Samyoun et al [71]	Smart wrist devices	ECG, EDA, EMG, TEMP, and RESP	RF, Extra Trees (EXT), DT, LDA, LR^ah^, and MLP^ai^	Accuracy and *F*_1_	Chest better than wrist sensors, and a combination of both is better than just chest.	LOOCV
Vidal Bustamante et al [32]	Wearables, wristband actigraphy data, and smartphone-based self-report surveys.	Self-report surveys on physical health, daily consumption habits, positive and negative affect, studying behaviors, stress levels and sources, sociability and support, and actigraphy	Linear modeling and clustering	BIC^aj^	—	—
Wu et al [74]	Empatica E4	EDA, BVP, and HR	K-means model with 2 clusters	Silhouette score	Comparable to state-of-the-art unsupervised methods.	—
Tutunji et al [76]	Empatica E4	HR, SC, ST^ak^, ACC, and surveys	Linear mixed-effects models, paired sample *t* test, and RF	Error rate	Individualized models combined EMA^al^ with physiology performed best, while group-based models performed worse.	LOSO^am^ and LOBO^an^
Abdul Kader et al [78]	Empatica E4	ACC, BVP, TEMP, EDA, HR, and HRV^ao^	DNN^ap^	Accuracy, precision, recall, *F*_1_-score, and AUROC^aq^	Privacy-preserving stress detection system using federated learning, providing privacy to the patient’s data.	CV
Vos et al [29]	Empatica E4, Mobi, and RespiBAN	EDA, HRV, ECG, ACC, EDA, ST, HR, SPO2^ar^, ACC, BVP, IBI^as^, EMG, and RESP	RF, SVM, ANN, and XGBoost	Accuracy, precision, recall, and *F*_1_-score	An ensemble ML^at^ model trained on a synthesized multidataset to improve the generalization of prediction.	LOSO
Darwish et al [82]	Fitbit Sense 2, Flowtime, Movesense, Prana, and Sentio Solutions Feel Terapeutics	ECG, EDA, and RESP	RF, XGBoost, KNN, LR, DT, AdaBoost, Extra Trees, Bagging classifier, LDA, and QDA^au^	Accuracy, precision, recall, and *F*_1_-score	Validated multimodal wearable data in controlled (WESAD)^av^ and real-life (SWEET)^aw^ datasets for binary and 5-class stress detection.	CV
Bloomfield et al [3]	Oura Ring	Sleep, surveys, ACC, HR, HRV, and RESP	Mixed-effects regression models	Coefficient and *P* value	Used sleep estimates from wearables in the prediction of perceived stress.	—
Nazeer et al [84]	Customized proposed STRESS-CARE and stress detection sensor	ECG, EDA, BVP, EMG, TEMP, and sweat	XGBoost, DT, RF, and SVM	Accuracy and *F*_1_-score	Wrist-worn sensors (2-class and 3-class) prediction model performed worse than chest sensors (2-class).	Exploring various combinations of input sensor data.
Xuanzhi et al [88]	Empatica E4 and RespiBAN	EDA and HRV	Attention mechanism-based XLNet model, BrainNet, Xception, EfficientNetB4, VGG19, ResNet-50, MobileNet, and InceptionV3	Accuracy, recall, precision, and *F*_1_-score	Proposed attention mechanism-based XLNet model for continuous stress monitoring.	Train/test split and CV
Vidal et al [89]	Actigraphy	Sleep duration and self-reports on stress and sleep	Individual-level linear model with a Bayesian framework	Bayesian metrics (pd, UIs, ROPE, ESS, and R-hat)	Negative associations between sleep duration and perceived stress in participants.	Stable estimates of lead-lag associations.
Tazarv et al [91]	Samsung Galaxy Gear Sport	PPG, ACC, GYR^ax^, and atmospheric pressure	SVM, XGBoost, and RF with a context-aware Deep Q-Network (DQN)	Recall	A model with a context-aware active learning strategy for fine-grained, personalized stress detection worked with fewer queries.	LOSO
Ganesan et al [97]	Empatica E4	ACC, PPG, ECG, EMG, EDA, RESP, and TEMP	DNN and 1D-CNN	ROC-AUC^ay^, *F*_1_-score, accuracy, latency, and memory	An optimized, cost-effective, real-time, and energy-efficient DNN model demonstrated superior performance.	—
Neigel et al [99]	Oura Ring	HR, HRV, activity, and sleep	Mixed effects model	*P* value and regression coefficients	Heightened waking HR and max waking HR, alongside sleep HR, sleep HRV, activity patterns, and sleep phases, during periods coinciding with significant academic and societal events.	—
Pogliaghi et al [100]	Empatica E4	EDA and BVP	RF, XGBoost, and MTL	*F*_1_-score and accuracy	The proposed MTL model improved compared to single-task models.	LOSO
Lopez et al [105]	Fitbits	Calories burned, HR, sleep, steps, and distance	AdaBoost	*F*_1_-score	Aggregation levels of 4 and 12 hours performed best with the calories and sleep modalities outperforming other modalities.	LOSO
Wilfred et al [106]	Wyoware devices	EMG and GSR^az^	Transfer learning model networks with CNN compared with SVM, DNN, LSTM^ba^, and CNN + LSTM	Accuracy, precision, recall, and *F*_1_-score	The proposed stress detection tool, equipped with an IoT^bb^ system and VR^bc^, worked best.	—
Gaitán-Padilla et al [108]	customized wearable polymeric optical fiber sensor, fiber Bragg grating, and ECG sensor	Pulse and RESP	Bagged DT, KNN, DT, and SVM	Accuracy, precision, recall, and *F*_1_-score	Used a low-cost wearable polymeric optical fiber sensor to classify stress.	Comparison
Gupta et al [109]	Empatica E4 and RespiBAN	ECG, PPG, and GSR	RF, SVM, LDA, KNN, NN, and DT	Accuracy, sensitivity, specificity, precision, *F*_1_-score, Matthew’s correlation coefficient, and Cohen kappa	Wrist-worn sensors performed less than chest-worn sensors.	LOOCV
Sakanti et al [112]	RespiBAN	ACC, ECG, EDA, EMG, TEMP, and RESP	Extreme gradient boosting	Accuracy	Evaluated extreme gradient boosting in stress classification with high accuracy.	—
Shedage et al [113]	Empatica E4 and RespiBAN	BVP, ECG, EDA, EMG, RESP, TEMP, and ACC	LR, DT, RF, and SEL^bd^	Accuracy	SEL worked for a generalized, personalized model. SEL: LR, DT, and RF as base model and RF as meta model.	—
Tanwar et al [115]	Empatica E4 and RespiBAN	ECG, EDA, EMG, ACC, TEMP, and RESP	XGBoost, LGBoost^be^, and CatBoost^bf^	Accuracy	Evaluated the effectiveness of data fusion methods, an accuracy increases with increase in modalities, and 5 modalities had best performance.	Train/test split
Gullapalli et al [116]	PPG sensors in consumer-grade earbud devices	HRV	RF	Accuracy, specificity, and sensitivity	Compared stress detection with the most prominent HRV library HeartPy.	—
Parousidou et al [119]	Empatica E4 and RespiBAN	ECG, EDA, EMG, ACC, TEMP, and RESP	LDA, log reg, DT, NB^bg^, RF, GB, user-based splitting, single-attribute splitting, multiattribute splitting, single task learning, and MTL.	*F*_1_-score	Personalized approach performed better in lab settings and worse in the wild, outperforming one-size-fits-all.	—
Sethia et al [121]	Empatica E4	IBI from HRV, BVP, EDA, and TEMP	GB, RF, DT, SVM, KNN, and XGBoost	Accuracy	EDA + BVP + HRV performed well with GB for 2-level and 3-level stress classification, with HRV and EDA being the most important features.	—
Benita et al [123]	Empatica E4	PPG	CNN	Accuracy	Developed a stress detection system investigating CNN.	Train/test split
Carmisciano et al [125]	Empatica E4 and RespiBAN	EDA and HR	FDA^bh^, RF, and LM^bi^	Partial R-squared	FDA models generally fit better than LM and RF.	—
Warrier et al [126]	RespiBAN	ECG, EDA, EMG, RR, TEMP, and ACC	DNN and federated learning	Accuracy	Federated learning–based stress detection method, focused on privacy protection with high accuracy.	Train/test split
Hoang et al [1]	Empatica E4 and RespiBAN	ECG, EDA, EMG, ACC, TEMP, and RESP	XGBoost	*F*_1_-score, precision, and recall	Personalization performed better	Train/test split
Hasanpoor et al [129]	Empatica E4	PPG	CNN	Accuracy	Optimized model of reduced size and space addressing resource constraints.	Train/test split
Tanwar et al [132]	Empatica E4 and RespiBAN	ECG, EMG, and RESP	A hybrid deep learning network consisting of long short-term memory and gated recurrent unit (LSTM-GRU) with an attention layer	Accuracy	Proposed well-performing personalized stress detection.	—
Huang et al [133]	RespiBAN	ECG	A hybrid model combining CNN and SVM	Accuracy	A hybrid model combining a CNN and SVM performed with high accuracy.	Train/test split
Oh et al [134]	RespiBAN	ACC, ECG, EDA, EMG, TEMP, and RESP	Three CNN-based classifiers and an ensemble attention module	Accuracy	An ensemble-based stress detection model that used multimodal features and metadata to capture personalized patterns.	Train/test split
Thapa et al [135]	Empatica E4 and RespiBAN	ECG, EDA, EMG, ACC, TEMP, and RESP	Conducted experiments using 4 state-of-the-art LLMs:^bj^ GPT (4 and 3.5-Turbo), Llama2, BioMistralDARE, and Gemini-Pro.	Accuracy and MAE	For LLMs, parameter size did not correlate with accuracy; smaller models such as GPT-3.5-Turbo performed comparably to larger ones like GPT-4, though these models overall performed worse.	—
Tsiampa et al [137]	Empatica E4	EDA	Statistical correlation analyses	Correlation	A relationship exists between EDA and stress levels related to social media content, with a strong correlation.	—
Fazeli et al [138]	Garmin vivoactive 4S	HR, HRV, number of floors climbed, BMR^bk^ kilocalories, distance traveled, activity levels, SPO2, and RESP	RNN, LSTM, and MLP	Accuracy	Proposed a multimodal semisupervised framework for tracking physiological precursors of the stress response; Late-fusion + Supervised Training + Contrastive Regularization performed best.	—
Subathra and Malarvizhi [139]	Empatica E4	EDA and HR	K-means and agglomerative clustering	Silhouette score	Agglomerative clustering obtained in the proposed method outperformed.	—
Andreas et al [141]	Empatica E4 and RespiBAN	ECG, EDA, EMG, ACC, TEMP, and RESP	CNNs in conjunction with transfer learning	Accuracy	Proposed method’s effectiveness outperformed state-of-the-art classification techniques in the field using transfer learning.	—
Lee et al [21]	Empatica E4 and RespiBAN	ECG, EDA, EMG, ACC, TEMP, and RESP	DNN augmented with attention mechanisms	Accuracy	Enhanced DNN capabilities by integrating both raw signals and human-engineered features altogether.	LOSO
Kasnesis et al [142]	Empatica E4 and RespiBAN	ECG, EDA, EMG, ACC, TEMP, and RESP features extracted by a temporal CNN.	TranSenseFuser is comprised of temporal convolutions followed by feature-level or sequence-level multihead attention.	Accuracy and *F*_1_-score	Model performed well for stress prediction.	LOSO
Ciharova et al [143]	VU-AMS	ECG and EDA	Bayesian ridge regression	Accuracy, *F*_1_-score, and r2	Performance ranged from acceptable to good, but only for the presentation stressor, best algorithm performance was a weak relationship between the detected and observed score	LOSO
Darwish et al [144]	RespiBAN	ECG, EDA, and RESP	RF, XGBoost, KNN, LR, DT, AB^bl^, ET, BAG^bm^, QDA, LDA, and ensemble models using majority voting and weighted averaging.	Accuracy	Binary stress classification performed better than five-class classification	K-fold CV
Nuamah [145]	Empatica E4 and Tobii Pro Glasses 2	Vagally mediated heart variability measures (vmHRV) and task-evoked pupillary response (TEPR)	Mixed-effects modeling	r2	vmHRV measures and TEPR are sensitive enough to quantify psychophysiological responses to recurrent task-induced stress	—
Saylam and İncel [19]	Fitbit	Step counts, active minutes, HR, and sleep metrics	RF, XGBoost, LSTM, and regression	MAE	With MTL, RF had the lowest error while looking back 7 and 15 days	—
Sa-nguannarm et al [146]	Empatica E4 and RespiBAN	ECG, EDA, EMG, ACC, TEMP, and RESP	Bi-LSTM	Accuracy and *F*_1_-score	The human lifelong monitoring model Bi-LSTM for stress behavior recognition performed well.	Train/test split
Nelson et al [147]	Smartphone	PPG	Mixed-effects modeling	r2	Smartphone-based PPG significantly covaries with self-reported stress and anxiety.	—
Dahal et al [148]	RespiBAN	HRV	RF	Accuracy	Identified person-specific stress events with an accuracy higher than 99% after a global training framework.	15-fold CV
Jiao et al [150]	PL3516 Powerlab 16/35 with TN1012/ST Pulse Transducer	PRV^bn^	SVM model with linear and radial basis function kernel	Accuracy	Developed a pulse rate variability detection model with RFE^bo^ feature selection.	5-fold CV
Belwafi et al [23]	EEG^bp^ sensor	EEG	Statistical thresholding mechanism on EEG bands	Accuracy, precision, recall, and *F*_1_-score	Proposed statistical thresholding mechanism on EEG bands approach achieved an average accuracy of 88.89%.	—
Patane et al [152]	Smartphone	Phone call duration, conversation, physical activity, app usage, and academic deadlines	RNN, Bi-LSTM, transformer with prompt tuning	MAE and MSE	Personalized mental well-being monitoring with RNN, Bi-LSTM, and transformer with prompt tuning, where prompt-based adaptation achieved lower prediction error.	Train/validation/test split a 70%-10%-20% ratio.
Subathra et al [153]	Custom-built wrist device	HR and EDA	Bi-LSTM	Accuracy and *F*_1_-score	Developed a wearable band, in Bi-LSTM, got F1-score of 99.38% and 98.88% in multiple datasets.	Train/validation/test split a 70%-10%-20% ratio.
Li et al [25]	PPG sensor	DASS-21^bq^ stress score, PRV, and dPPG	1DCNN-Bi-LSTM, cross-attention, and XGBoost	MAE and RMSE^br^	Analysis found fusion of PRV and dPPG signals yielded best detection performance.	5-fold CV
Van der Mee et al [154]	Garmin smartwatch	Garmin HRV-derived stress score and mood EMAs.	Firstbeat analytic algorithms, mixed-effects regression, logistic multilevel models, and ANOVA	AUC and statistical significance	Analysis found Garmin Stress Score was associated with high- and moderate-intensity positive mood; it was not associated with states of high arousal negative mood.	Statistical association analysis
Rosenbach et al [24]	Garmin Vivosmart 4 and Polar H10 chest strap	Garmin stress score, HRV, and HR	Linear mixed effect model	Statistical significance	Analysis found HR showed the strongest association with self‐reported stress, while the Garmin stress score demonstrated only marginal predictive value.	Statistical association analysis

a ECG: electrocardiogram.

b HR: heart rate.

c ANN: artificial neural network.

d SOM: self-organizing map.

e SOFM: self-organizing feature map.

f Not available.

g SC: skin conductance.

h TEMP: temperature.

i ACC: accelerometer.

j MAE: mean absolute error.

k SAM: Self-Assessment Manikin.

l EDA: electrodermal activity.

m EMG: electromyography.

n RESP: response.

o BVP: blood volume pulse.

p CNN: convolutional neural network.

q CV: cross-validation.

r PPG: photoplethysmography.

s SVM: support vector machine.

t Res-TCN: residual temporal convolutional network.

u LOOCV: leave-one-out cross-validation.

v MTL: multitask learning.

w MSE: mean squared error.

x XGBoost: extreme gradient boosting.

y KNN: k-nearest neighbor.

z DT: decision tree.

aa RF: random forest.

ab Ada-boosting: adaptive boosting.

ac NN: neural network.

ad LDA: linear discriminant analysis.

ae OMDP: optimized model decision process.

af GRU: gated recurrent unit.

ag RNN: recurrent neural network.

ah LR: logistic regression.

ai MLP: multilayer perceptron.

aj BIC: Bayesian information criterion.

ak ST: skin temperature.

al EMA: ecological momentary assessment.

am LOSO: leave-one-subject-out.

an LOBO: leave-one-batch-out.

ao HRV: heart rate variability.

ap DNN: deep neural network.

aq AUROC: area under the receiver operating characteristic curve.

ar SPO2: peripheral capillary oxygen saturation.

as IBI: interbeat interval.

at ML: machine learning.

au QDA: quadratic discriminant analysis.

av WESAD: Wearable Stress and Affect Detection.

aw SWEET: Stress in the Wild and Everyday Environment.

ax GYR: gyroscope.

ay ROC-AUC: receiver operating characteristic–area under the curve.

az GSR: galvanic skin response.

ba LSTM: long short-term memory.

bb IoT: internet of things.

bc VR: virtual reality.

bd SEL: stacked ensemble learning.

be LGBoost: Light Gradient Boosting Machine.

bf CatBoost: categorical boosting.

bg NB: naive Bayes.

bh FDA: functional data analysis.

bi LM: linear model.

bj LLM: large language model.

bk BMR: basal metabolic rate.

bl AB: adaptive boosting.

bm BAG: bootstrap aggregating.

bn PRV: pulse rate variability.

bo RFE: recursive feature elimination.

bp EEG: electroencephalogram.

bq DASS-21: Depression Anxiety Stress Scale–21 item.

br RMSE: root mean squared error.

### Critical Appraisal of Individual Sources of Evidence

Although critical appraisal is not required for scoping reviews, we conducted an assessment of study quality to better contextualize the strengths and limitations of the included evidence. To address the quality of each paper, we scored every paper across 4 categories on a scale from 0 to 2 as described in Multimedia Appendix 2 and shown in Multimedia Appendix 3. Given the diverse study designs among the extracted papers, we adopted a methodology similar to that used by De Angel et al [155]. This approach integrates the AXIS appraisal tool [156] for cross-sectional studies with the Newcastle-Ottawa Scale [157] for longitudinal studies. Papers were assessed using a 3-point scoring system: 2 points for fully meeting the criteria, 1 point for partial fulfillment, and 0 points for nonfulfillment.

Effect measures extracted from the included studies consisted of accuracy, *F*_1_-score, sensitivity, specificity, precision, recall, and other performance metrics reported for stress detection. These measures were used to compare model performance across studies. For population characteristics, the mean age and corresponding SDs were extracted whenever available.

### Synthesis of Results

Due to differences in study designs, methodologies, and outcome reporting, results were synthesized descriptively. Key study characteristics, signals measured, algorithms used, and sensor types were organized into structured tables to enable comparison across studies. Frequencies of the most commonly measured signals, best-performing algorithms, and most-used sensors were calculated and visualized using bar plots. Missing summary statistics were extracted as reported, with no additional transformations applied. No meta-analysis, subgroup analysis, or meta-regression was conducted; instead, the synthesis focused on identifying overarching trends across the included studies. Because the focus of this review was to characterize stress detection methods used among college-aged populations, we extracted data elements that were directly relevant to the review objectives, including participant characteristics, sensor types, physiological signals, analytical methods, and model performance outcomes. Broader intervention-related data items (eg, intervention protocols, adverse event reporting, and clinical outcome metrics) did not apply to the observational and experimental studies included in this review. Therefore, the extraction approach was intentionally streamlined to ensure consistency, interpretability, and comparability across heterogeneous study designs. In addition, we developed an evidence gap map to conceptually organize and summarize the literature across study conditions, methodological enablers, analytical approaches, barriers, and outcomes, highlighting recurring patterns as well as persistent gaps, following a prior standardized method [158].

### Ethical Considerations

This study is a systematic review of previously published literature and did not involve the collection of primary data from human participants. No new data were generated, and no individuals were directly recruited, observed, or intervened upon as part of this research. Accordingly, a formal review by an Institutional Review Board or Research Ethics Board was not sought. This determination is consistent with standard guidance that systematic reviews relying exclusively on publicly available, previously published data do not constitute human participant research requiring ethics board oversight.

All included studies were previously published in peer-reviewed journals and were assumed to have undergone appropriate ethical review by their respective authors and institutions before publication. No personally identifiable information was accessed, extracted, or reported at any stage of this review. The conduct of this review adhered to the ethical principles outlined in the World Medical Association Declaration of Helsinki and complied with applicable institutional, regional, and international standards for research integrity.

## Results

### Selection of Sources of Evidence

**Figure 1.**
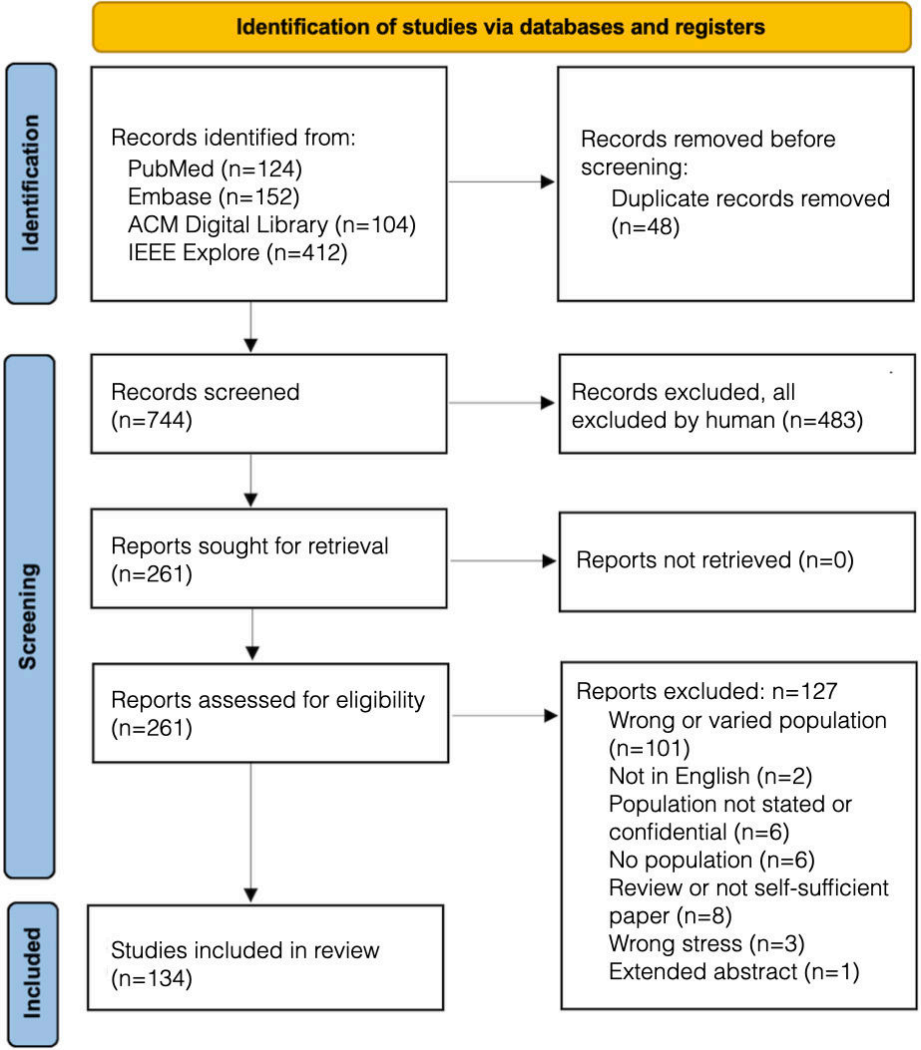
PRISMA (Preferred Reporting Items for Systematic Reviews and Meta-Analyses) flow diagram for study selection from medical and computer science databases.

Records were screened from IEEE Xplore, ACM Digital Library, Embase, and PubMed, with most records coming from technical journals. A total of 134 studies were included in the review out of the original 792 records, as illustrated in Figure 1 and Multimedia Appendix 4. Forty-eight records were removed after deduplication. Of the remaining records, 483 were excluded after 744 abstracts were screened for relevance. In total, 127 records were excluded after 261 full texts were screened for relevance and correct population. Summary characteristics of the final 134 included studies are provided in Figure 1 and Table 1.

### Demographic and Geographic Characteristics of Included Studies

Our population of interest included college-aged students aged 18‐24 years. In terms of sex demographics, about 72.4% (97/134) of studies specified the number of participants who were male, female, or nonbinary. Among the selected studies, 2 of 134 (1.5%) studies [39,137] failed to mention a sample size. Across the studies that reported sex distribution, most had a higher proportion of male participants than female, indicating a demographic imbalance that may limit the generalizability of findings. In terms of racial demographics, in 42 papers published from 2020 to 2022, about 9.5% (n=13) of papers included race distribution across their sample population, and 21 (50%) studies included other relevant health information, including preexisting conditions, mental health, and underlying illnesses. From papers published from 2020 to 2025, 26 (19.4%) studies were conducted in Europe, 14 (10.4%) studies were conducted in Asia, 2 (1.5%) studies were conducted in the Middle East, 21 (15.7%) studies were conducted in the United States, 3 (2.2%) studies were conducted in South America, and other studies did not explicitly mention where they were conducted. The higher number of studies conducted in Europe and the United States compared to Asia and other regions suggests regional variations in digital health adoption, research funding, and accessibility of wearable technologies. These differences may influence trends in stress detection research, highlighting the need for region-specific digital health strategies to address varying technological infrastructures, health care priorities, and user needs.

### Study Design and Data Collection Characteristics

More than half (62.8%, n=134) of the studies used preexisting datasets to implement their method of stress measurement. The rest of the studies were experimental in nature and carried out “de novo” data collection. Seventeen studies [32,45,46,54,57,61,65,72,73] were longitudinal in nature, published from 2020 to 2022, and 10 studies [3,76,82,89,91,95,99,105,152,154] were longitudinal in nature, published from 2023 to 2025, meaning data were collected for the same study population over a period rather than collected at one time point cross-sectionally. These longitudinal data consist of repeated observations at the individual level rather than data collected at multiple time points across different populations. Individual-level effects are confounded with cohort effects in cross-sectional studies, so being able to isolate and study the effect of time as a repeated measure is critical. Of these longitudinal studies published from 2020 to 2022, 2 were clear in addressing how they handled missing data. These studies either imputed missing values with each person’s channel-wise mean values of the day, where days with >25% sensor data missing were discarded [45], or removed missing data [57]. It is difficult to collect comprehensive, complete data from sensors longitudinally, where data are not always complete for each participant. About 6.6% (n=9) of studies included a recruitment method for participants. Two studies used volunteers, and 1 study invited participants to participate.

### Approaches in Stress Detection Research

**Figure 2.**
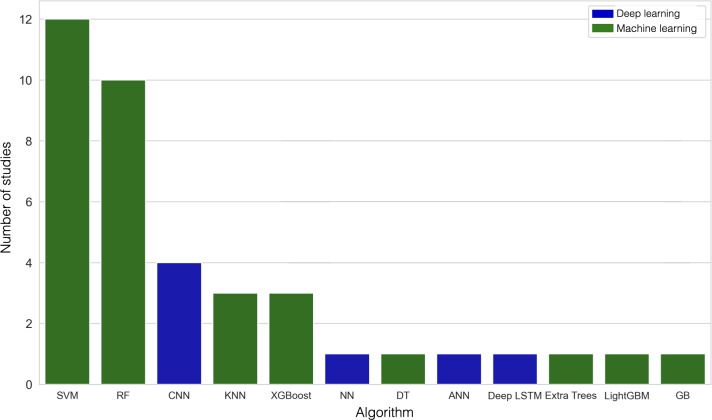
Best-performing algorithms across 36 studies comparing established methods. ANN: artificial neural network, CNN: convolutional neural network, DT: decision trees, Extra Trees: extremely randomized trees, GB: gradient boosting, KNN: k-nearest neighbor, LightGBM: light gradient boosting machine, LSTM: deep long short-term memory, NN: neural network, RF: random forest, SVM: support vector machine, XGBoost: extreme gradient boosting.

The extracted studies were classified into 3 primary methodological categories: algorithm comparisons (shown in Table 2), the development of custom stress measurement frameworks, and statistical analyses (illustrated in Table 3). Studies focusing on algorithm comparison primarily used 2 approaches: machine learning models, such as support vector machines (SVMs), random forest (RF), k-nearest neighbors, and extreme gradient boosting (XGBoost), which used handcrafted features for stress detection, or deep learning methods, such as convolutional neural networks (CNNs), to automatically extract relevant features [159]. Among the studies reviewed, SVM demonstrated the highest performance, with 33.3% (n=45) of papers identifying it as the best-performing algorithm, as illustrated in Figure 2. In comparison, 11.1% (n=15) of the studies reported CNN as the best-performing model [50,58,67,103]. One study evaluated 3 boosting algorithms—XGBoost, Light Gradient Boosting Machine, and CatBoost—tree-based ensemble methods that iteratively improve weak learners to enhance classification, evaluating the effectiveness of data fusion methods [115].

One paper [31] focused on comparing long short-term memory (LSTM) and a combination of LSTM and CNN. This study found LSTM alone to perform better. Two studies in Table 3 that focused on a single framework supported the use of a single modality or a modest set of signals [66,69]. Studies that focused on the comparison of chest and wearable devices found chest devices to perform better [84,109], but chest devices in combination with wrist devices performed the best [71]. Most of these studies focused on time-agnostic algorithms, as shown in Table 2. We also found studies using wrist wearables (eg, Empatica, Microsoft Smartband 2, Fitbit Charge 2, and Samsung Galaxy Gear Sport Watches) and chest-worn devices, with core physiological signals such as EDA, galvanic skin response, HR, photoplethysmography, HRV, respiration, or temperature, evaluated using k-fold cross-validation, leave-one-out cross-validation, or leave-one-subject-out evaluation, and reported performance metrics such as *F*_1_-score, accuracy, precision, and recall. In the “best” column, classic machine learning models were most often SVM, followed by RF, while deep learning wins were fewer (occasional CNN, deep neural network, and a single LSTM). Few studies in Table 2 incorporated nonphysiological or contextual signals [61,72,73]. Recent studies examining the association between sleep and stress have leveraged data from the Oura Ring [3,99]. Two recent studies using Garmin smartwatch–derived stress scores found significant associations with high- and moderate-intensity positive mood in 1 study [154], while another reported a stronger association of HR with self‐reported stress, and the Garmin stress score demonstrated marginal predictive value [24].

**Figure 3.**
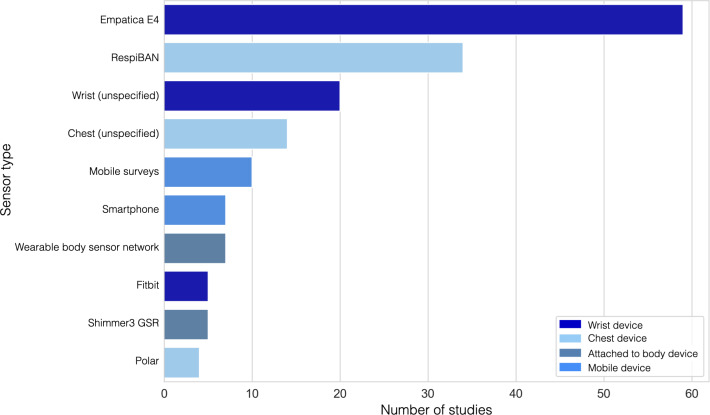
Top 10 sensors used across all 134 studies.

**Figure 4.**
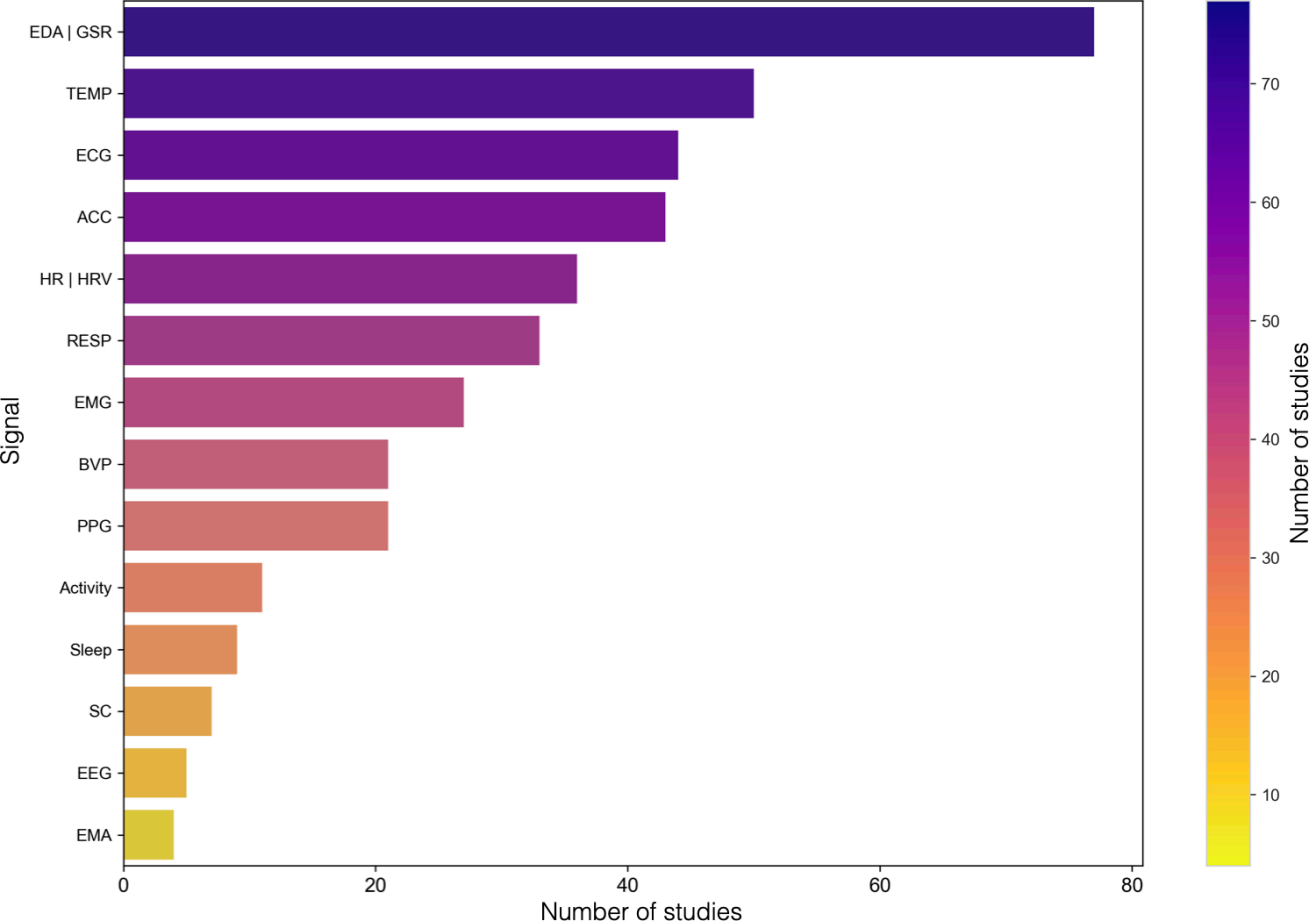
Distribution of top physiological signals used in reviewed studies, including ecological momentary assessment (EMA) as a self-report measure. Many studies used multiple signals, which are counted in the bar plot. ACC: acceleration, BVP: blood volume pulse, ECG: electrocardiography, EDA: electrodermal activity, EEG: electroencephalogram, EMG: electromyography, GSR: galvanic skin response, HR: heart rate, HRV: heart rate variability, PPG: photoplethysmography, RESP: respiration, SC: skin conductance, TEMP: temperature.

Studies mainly aggregated stress on a binary or 3-tier scale, meaning participants were either identified as stressed or not stressed, as opposed to being measured on a continuous scale. Here, a continuous scale captures stress fluctuation over time rather than binary or categorical labels. Sensors or tools used to measure physiological signals included various wrist, chest, and full-body sensors alongside mobile surveys. Figure 3 details the various devices used and shows that wrist sensors, in general, were the most widely used sensor type. About 72.4% (n=97) of the studies used well-validated stress tests or tasks for their models, such as the TSST [160], mental arithmetic tests, video stimuli, the Stroop color word test, startle response tests, cold-pressor tests, or public speaking, to reliably trigger stress responses while incorporating restful periods as a baseline [22]. About 8.3% (n=11) of the studies used self-reported SMS text messaging surveys in their supervised machine learning models. The various physiological features and signals measured are illustrated in Figure 4. The most common signal was EDA, appearing in 57.5% (n=77) of studies. Figure 4 shows the top signals measured per study, including instances where papers used multiple signals together.

### Most Commonly Used Wearable Stress and Affect Detection Datasets in Stress Detection

Of the 62.8% of studies that used some preexisting datasets, around 80% (n=67) used the Wearable Stress and Affect Detection (WESAD) dataset, for instance, including papers published from 2020 to 2022 [18,38,42,43,48,55,56,58,59,63,66-71] or a few published from 2023 to 2025 [2,28,75,79,86,94,103,113,122,141]. This dataset was commonly referenced in papers included in this review. This dataset is publicly available and is a widely used dataset for stress and affect detection [161]. The mean age of participants is 27.5 years with a SD of 2.4 years. The sample included 3 females and 12 males for a total of 15 participants. Heavy smokers and pregnant women were excluded, and the participants were composed of graduate students. The signals collected include physiological and motion data from chest-worn and wrist-worn devices. Measurements include blood volume pulse, ECG, EDA, electromyography, respiration, body temperature, and 3-axis acceleration. The protocol used elicits 3 emotional states: baseline, stress, and amusement, followed by a meditation phase. Benchmarks for comparison used the well-studied stress induction method, the TSST, with 0.93 accuracy and 0.91 *F*_1_-score for distinguishing stress, using a linear discriminant analysis classifier, using only chest-based physiological signals.

Although many papers used this same dataset, they experimented with different physiological signals as well as motion data when extracting features for modeling. Modeling and validation methods also varied. The algorithms with the best performance when applied to the WESAD dataset included SVM, RF, XGBoost, k-nearest neighbor, decision tree, deep neural network, self-supervised learning, artificial neural networks, large language models, and CNN. In addition to WESAD, recently published papers used other datasets, including SWELL [29], AffectiveROAD [81], VerBIO [96], S-TEST, or DS-3 [101].

### Quality Assessment of Included Studies

**Figure 5.**
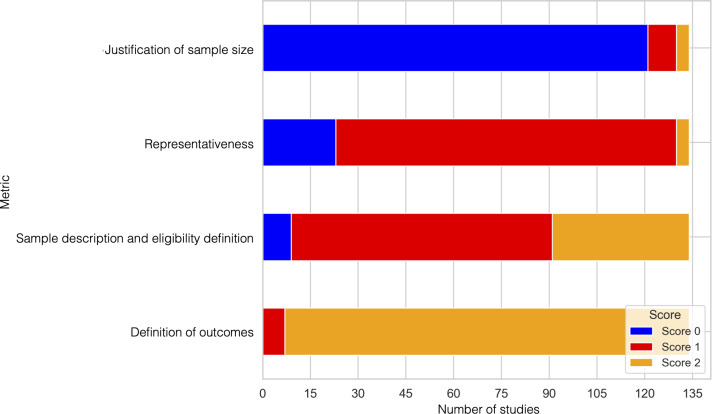
Quality of the literature in each domain. The figure shows the scoring across papers in each category from 0 to 2, with 0 indicating not fulfilled, 1 indicating partially fulfilled, and 2 indicating fulfilled.

Figure 5 shows a breakdown of quality score assessments for all extracted papers, broken down into 4 categories. Papers were scored 0, 1, or 2 for each category. An explanation of each category’s scoring is provided in Multimedia Appendix 2, and the individual score breakdown by category for each paper is provided in Multimedia Appendix 3. In general, outcomes and sample descriptions were clearly stated, with most papers having a quality score of 2. However, representativeness and justification of sample size were areas in which many papers did not perform as well. Representativeness was cited as a common issue across many papers, as samples were limited due to recruitment processes for participants or the data that were available. The samples were also limited by age due to the demographic of interest in this review. Around 27.6% (n=37) of papers failed to give sex demographic information. Most papers analyzed used experimental data from other sources or open-source, publicly accessible datasets such as the WESAD dataset, which did not justify the chosen sample size. From papers published from 2020 to 2022, only 2% of papers failed to give sample size information; however, sample size justification was rarely given, although the papers that did address this issue cited their voluntary recruitment process as a limitation. Almost none of the studies analyzed did a power analysis to determine sample size before running the stress studies, which is a major shortcoming. Across recent papers published from 2023 to 2025, almost all clearly defined outcomes and described their samples, but very few addressed representativeness, and only 3 papers [24,143,154] justified their sample size published, highlighting a major gap in methodological rigor.

**Figure 6.**
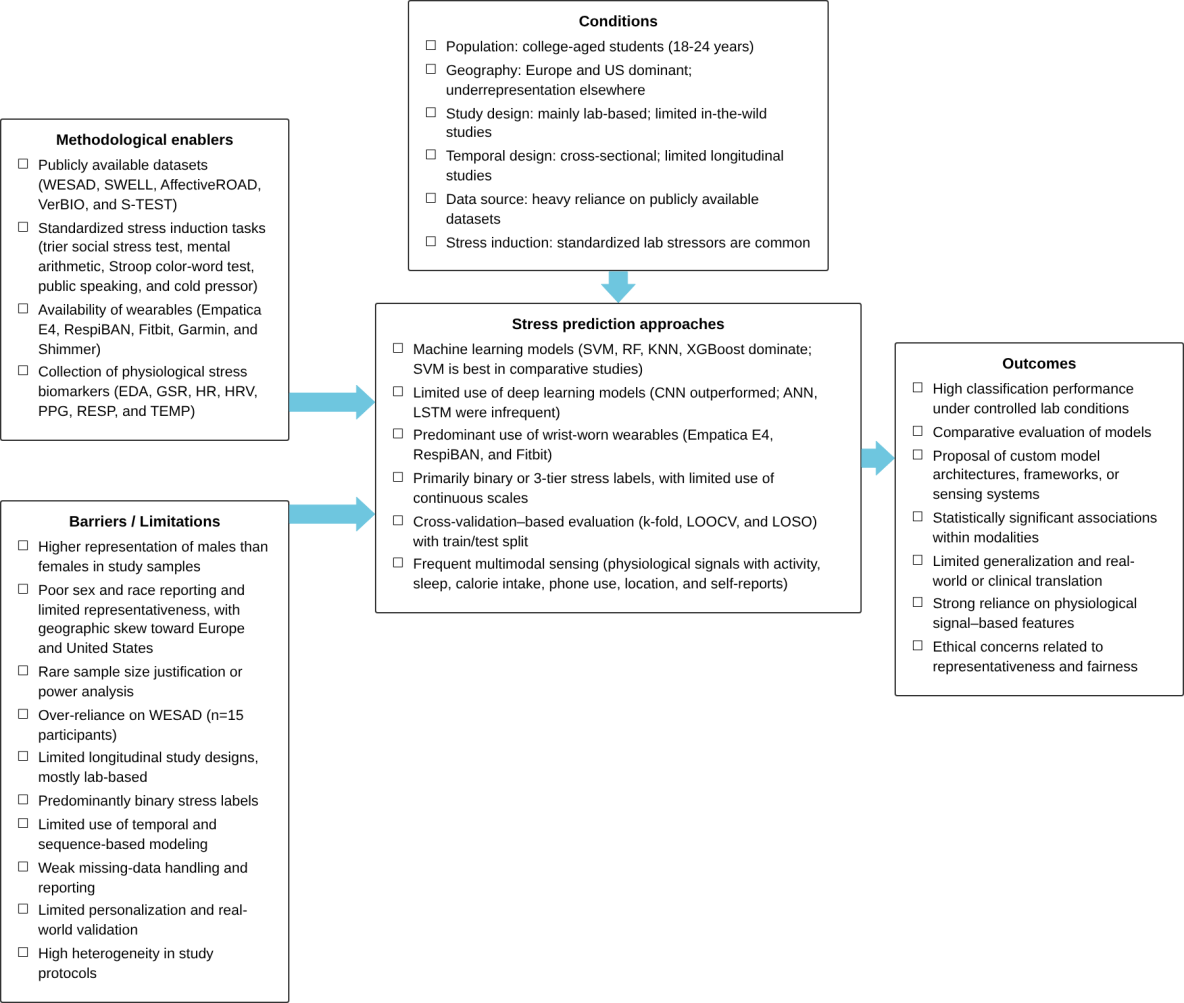
Gap map summarizing methodological enablers, study conditions, modeling approaches, barriers, and outcomes in wearable-based stress prediction studies among college students. ACC: acceleration, BVP: blood volume pulse, ECG: electrocardiography, EDA: electrodermal activity, EEG: electroencephalogram, EMA: ecological momentary assessment, EMG: electromyography, GSR: galvanic skin response, HR: heart rate, HRV: heart rate variability, PPG: photoplethysmography, RESP: respiration, SC: skin conductance, TEMP: temperature.

Finally, these findings point to substantial heterogeneity and a meaningful risk of bias across the included studies. The wide variation in sample sizes, inconsistent reporting of demographic characteristics, limited disclosure of health information, and strong geographic skew toward Europe and the United States contribute to structural differences that complicate direct comparison of results. This heterogeneity is further shaped by the heavy reliance on the WESAD dataset, a publicly available dataset with only 15 predominantly male participants, with a mean age of 27.5 years, which results in many studies concluding a small and demographically narrow sample. Such repeated use of a single dataset increases the likelihood that reported model performance reflects the characteristics of WESAD participants rather than capturing variability among college-aged students. Accordingly, the synthesized findings should be interpreted with caution, acknowledging that both heterogeneity in study design and risk of bias in sampling and reporting may influence observed performance patterns and limit the extent to which results can be generalized. Using a relational synthesis approach, Figure 6 presents an evidence gap map that synthesizes methodological enablers, study conditions, stress prediction approaches, barriers, and outcomes observed across the included studies. The map illustrates a research landscape shaped by publicly available datasets, standardized in-laboratory stress protocols, and widespread use of wrist-worn physiological sensors. At the same time, it highlights recurring constraints including a predominance of laboratory-based study designs, heavy reliance on publicly available datasets, and limited demographic representativeness. While many studies report strong classification performance using classical machine learning models under controlled conditions, comparatively fewer examine temporal stress dynamics, personalization, or real-world deployment.

## Discussion

### Overview

In this scoping review, we examined how stress is measured among college-aged students using wearable technologies and machine learning methods between 2020 and 2025, to identify commonly used wearables, the most informative physiological signals, and the best-performing algorithms. Across the literature, we found that SVMs among traditional machine learning models and CNNs among deep learning models were the strongest performers for stress classification. Wrist-worn devices were the predominant sensor platform, and EDA was the most frequently measured and most informative signal. However, most studies relied on small, homogeneous samples, frequently used controlled laboratory datasets such as WESAD, and commonly used binary (stressed vs not stressed) labeling approaches, raising concerns about representativeness and ecological validity. Our quality assessment further revealed inconsistent demographic reporting, insufficient justification of sample sizes, limited attention to social determinants of stress, and substantial variation in how psychological stress was defined, elicited, and validated across studies.

### Modeling Approaches for Stress Prediction

Regarding stress prediction model performance, the strong performance of SVMs can be attributed to their robustness in handling high-dimensional physiological data [33,144], their ability to generalize well by maximizing the margin between classes, and their effectiveness in small and imbalanced datasets, which are common in stress detection studies [162]. Additionally, the flexibility of SVM in using different kernel functions [163] allows them to model complex, nonlinear relationships in physiological signals without requiring deep feature extraction. These advantages likely contribute to their superior performance compared with other traditional machine learning models in stress classification. However, SVMs are computationally expensive and may not be practical for real-time applications [164]. More efficient and scalable approaches are needed to enhance practicality in the field. Deep learning models, particularly CNNs, outperformed traditional machine learning approaches in comparative analyses [82,85]. Although CNNs capture spatial patterns in temporal data, they do not have memory in their architecture, reducing their effectiveness on longitudinal temporal data [165], indicating a need for algorithms that explicitly model temporal patterns, such as RNNs [74]. One study focusing on the comparison of various machine learning and deep learning methods attempted to use a version of an RNN in the form of an LSTM. This paper reported the greatest performance with LSTM alone, as opposed to a combination of LSTM and CNN, indicating some value in noting and using temporal patterns. In addition, emerging evaluations of large language models for stress prediction [135] did not perform well and suggest that parameter count does not consistently correlate with performance. For example, GPT-3.5-Turbo performed comparably to GPT-4 on WESAD [109]. These findings indicate that identifying key biomarkers is essential for improving model efficiency [115]. From 2023 to 2025, published literature emphasized personalization and multitask learning to enhance stress-prediction performance and generalizability [70,79,98,107,112,127]. In addition, 1 study explored stress detection in a virtual reality environment integrated with an Internet of Things system, demonstrating the potential of immersive technologies for stress monitoring [85].

### Wearable Technologies and Physiological Signals

Wrist wearables were most commonly considered [166] as they seem less encumbering than full body or chest wearables [22] while attaining better measurement of physiological signals than surveys or smartphones. Other wearable sensors used across studies included chest wearables, full body sensors, or some combination of chest and wrist wearable signals. We saw that EDA was the most frequently measured signal across papers and is important in stress detection [167], since it provides valuable information about a person’s sympathetic nervous system activity, which is closely linked to emotional responses, including stress. Most papers used multiple signals in their model building, with EDA most commonly contributing to a more accurate model. For instance, building a stress detection model incorporating both HR and EDA [22,26,81] data might allow for a more comprehensive, accurate, and context-aware assessment of stress and other emotional responses. Ensuring the reliability and reproducibility of physiological measurements is crucial for real-world stress detection [26]. Variability in sensor accuracy, signal quality, and environmental factors can impact consistency [22]. Validating models across diverse settings improves generalizability and practical applicability [168].

### Conceptualizing and Measuring Psychological Stress

We saw that most studies used a binary model of stress in which an individual is identified as either stressed or not stressed. A few studies extended beyond binary classification by using multiclass stress prediction (eg, 3-class [62] or 5-class [59,125] models), which allows a somewhat finer-grained view but still treats stress as discrete states. There is a need for a model more in line with how human stress manifests, such as a continuous scale [26,169]. For example, an individual might feel mildly stressed, which is worth noting and which cannot be captured on a binary scale of stress [150]. On a binary scale, mild stress may be interpreted as either diminished or heightened stress. A continuous scale for stress monitoring is valuable for capturing individual differences and for understanding the dynamic nature of stress [150].

We found a lack of detailed explanations on how psychological stress was identified. Accurately distinguishing psychological stress from other physiological responses is complex, as HR alone is insufficient for stress detection [154]. For example, HR alone cannot reliably indicate stress, as an elevated HR may result from various factors [170], such as jogging or facing an unprepared mathematics test. A stress detection model based solely on HR data could misclassify natural variations in HR, such as those caused by excitement or physical activity during social events, as stress, leading to inaccurate assessments [169]. One critical detail to note in studies of stress is the differentiation between physiological and mental stress. This distinction is complicated for wearable devices [154]. To accommodate this, studies need to look at a participant’s resting data while they are confirmed to be stressed, as well as their accelerometer data, if necessary, to check movement patterns, and consider these factors while detecting significant stress moments [169]. One’s activity must be noted to clearly identify psychological stress. Many studies used some well-validated stress tasks to account for this concern, but could benefit from clearer explanations of how their stress tasks accommodate this issue. These stress tasks mostly used tests such as mental arithmetic, Stroop test, public speaking, or cold-pressor tests, with participants putting their hands in ice water, to benchmark stress [22,26]. By contrast, other datasets (eg, “A Wearable Exam Stress Dataset for Predicting Cognitive Performance in Real-World Settings” [124]) inferred stress levels indirectly from examination grades, raising concerns about the accuracy of stress labeling. Studies that did not incorporate a stress task often used self-report surveys to monitor whether someone is stressed [168,171]. Self-report measures often face challenges with accuracy and completeness [172]. While frequent and timely survey prompts can improve accuracy, they do not fully address issues of completeness. Additionally, repeated survey checks may increase participant burden, potentially leading to survey fatigue and lower response rates [173]. There is also a need for better transparency regarding the wording of questions and the frequency of surveys to ensure consistency and minimize bias [174].

### Concerns Related to Study Design and Reporting

When analyzing the quality of research, we saw a need for larger sample sizes [175]. Larger sample sizes help reduce bias, provide a better representation of the target population, and lower the impact of outlier participants [176]. We observed that many studies relied on the WESAD dataset [177], a widely used dataset for stress and affect detection. However, WESAD includes only 15 participants, making it a limited representation of the college student population. Additionally, since WESAD data were collected in a controlled laboratory setting, they do not reflect real-world (“in the wild”) stress detection, where external factors and daily life variability play a significant role [171,178,179]. In fact, 1 study that used WESAD achieved strong performance under laboratory conditions but failed to generalize effectively in real-world settings [119], further underscoring the limitations of laboratory-based datasets.

Many studies did not report racial or ethnic demographics or have a representative sample regarding sex. This was a commonly identified issue within papers, as many samples relied on volunteers. Many papers also failed to report on other demographics of their samples besides sex or ethnicity, such as populations for exclusion. This includes excluding populations taking certain medications, populations with certain mental health histories, populations engaging in drug use, or pregnant populations. Knowing the populations for exclusion is crucial for replicability and transparency, as well as for bias detection and interpretation of results [180-182]. Although our population of interest was students, there is a need for more varied student demographics in samples regarding sex, race, and ethnicity, capturing different social determinants [183]. Given that stress is influenced by various social determinants [184,185], future studies should incorporate factors such as socioeconomic status, neighborhood context, physical environment, racial minority representation, and health-lifestyle interactions [186]. Including these elements would provide a more comprehensive understanding of stress in college students. One paper mentioned that its sample may not be representative because participants were recruited from an elite, private university [32]. Along these lines, there is a need for better justification of sample selection as well as sample size. Finally, missing data present a significant challenge in stress studies, affecting both comparability across studies and the reliability of findings [187]. The way missing data is handled, whether through imputation, exclusion, or other techniques, can influence study outcomes and lead to biased conclusions [188]. There is a need for more complete data and more detailed descriptions of how missing data were handled, particularly in longitudinal studies [189].

### Relationship to Prior Reviews and Contribution of This Work

Prior literature reviews have explored various aspects of stress detection using wearable technology and machine learning. A meta-analysis examined the effectiveness of wearable AI in diagnosing and predicting stress among students, while emphasizing the need for real-world validation and improvements [190]. Another review categorized stress detection approaches based on different wearable sensor types and environments such as driving, studying, and working [191]. A separate study systematically assessed biosignal responses to psychological stress, analyzing electroencephalogram, ECG, EDA, HRV, respiration, and temperature to evaluate their reliability and consistency [192]. A prior review also examined machine learning techniques used in stress monitoring research, focusing on model generalization when training on public datasets [20]. Another review focused on wearable technologies and smart devices for detecting depression, anxiety, and stress, discussing physiological markers such as HRV, EDA, and electroencephalogram, along with their market availability [193]. Finally, a review analyzed physiological parameters such as HR, temperature, humidity, blood pressure, and speech, exploring various stress detection sensors and machine learning-based classification techniques [194]. Our scoping review extends this literature by specifically focusing on stress measurement in college-aged students, reviewing recent papers published from January 2020 to December 2025, analyzing common datasets, sensor types, and the best-performing machine learning algorithms used in research. We also evaluate weaknesses in current methodologies through a quality assessment while identifying best practices in study design, feature selection, sensor use, and algorithmic approaches.

Taken together, the findings of this scoping review highlight that progress in wearable-based stress detection for college-aged students [3,32,46,73] is constrained primarily by methodological and conceptual design choices rather than sensor availability for digital phenotyping of stress [195] or algorithmic capacity [18,28,30]. While multimodal physiological sensing, particularly EDA combined with cardiac measures, shows consistent promise [22,26], the field remains highly reliant on small, controlled datasets such as WESAD [177] and binary stress formulations that fail to capture the continuous [26,169], context-dependent nature of stress in students’ daily lives [171]. Advancing this area will require a shift toward larger [175], more diverse cohorts that reflect different social determinants of health [186], and real-world datasets that support generalizable human behavior modeling [168,196]; along with transparent reporting of participant characteristics, exclusion criteria, and missing data handling [189]; and modeling approaches that explicitly account for temporal patterns [95], personalization [1], and contextual information from students’ behavioral patterns [152]. These improvements are not only methodological but also ethical, and without representative samples and robust validation in real-world settings, stress detection systems might risk reinforcing bias [197] and producing misleading inferences when deployed in student populations [183]. By synthesizing recent evidence and identifying persistent gaps, this review provides a foundation for designing more reliable, interpretable, and equitable stress monitoring systems that can support just-in-time interventions and inform institutional strategies to improve student mental health [5].

### Limitations

Our focused and systematic approach targeting stress in college students in recent years allows for a more detailed analysis. Recency allows for analysis of the most up-to-date and commonly used sensors as well as the newest algorithms. By systematically categorizing the approach taken by each study, along with the devices used and signals measured, we can synthesize the information, establish trends, and make conclusions about best-performing methods and practices. Many studies relied on commonly used datasets, such as WESAD. Using the same dataset across different research projects enables benchmarking, allowing for direct comparison of methodologies and an understanding of why results may vary across approaches. A common challenge in the reviewed papers was the inclusion of multiple populations or datasets within a single study. While our primary focus was on college students, some papers analyzed mixed populations or multiple datasets. However, as long as college students were included, these studies were still considered in our review. Many papers also used overlapping datasets such as the WESAD dataset, although different papers used different parts of the dataset along with different models. This may lead to some redundancy in findings. The commonly used dataset, WESAD, with only 15 participants, had limited sample sizes, introducing potential bias and reducing the likelihood of capturing a truly representative population. Additionally, only studies published in English were included, as this was the language accessible to our reviewers, which may have led to the exclusion of relevant research.

### Conclusions

This scoping review provides a focused synthesis of wearable- and digital tool–based stress detection research specifically among college-aged students, a population often overlooked or aggregated with broader adult samples in prior reviews. Current research highlights the need for larger and more diverse samples to improve representativeness, as many studies rely on a limited number of existing datasets, potentially leading to overlapping findings. Greater diversity in sex and ethnic demographics, along with clearer justification of sample sizes and improved demographic reporting, is essential for understanding population-level stress patterns. Methodologically, most studies conceptualized stress as a binary state (stressed vs not stressed), failing to capture variations in intensity, such as mild or moderate stress that can be chronic and clinically meaningful. Few studies used algorithms such as RNNs, which can capture temporal patterns, despite the importance of tracking stress progression over time. Greater emphasis on time-dependent modeling could enhance the understanding of how stress evolves. Many studies failed to clearly distinguish between psychological stress and physiological stress responses, despite the critical need for distinct measurement approaches. More precise definitions and methodologies are necessary to differentiate between these 2 aspects of stress effectively. In real-world settings, these limitations constrain the generalizability and clinical usefulness of stress detection systems.

To strengthen the credibility and generalizability of future research, studies should provide clear justifications for their sample sizes and, where possible, aim to recruit larger cohorts that reduce bias and improve statistical reliability. The field would also benefit from the development and use of more varied datasets, which can limit overlap across studies and reduce potential sources of bias. Increasing diversity in participant recruitment is essential; researchers should ensure representation across race, sex, socioeconomic status, and environmental contexts, as well as variation in behavioral and lifestyle factors such as sleep duration and efficiency, physical activity, phone usage, social media engagement, and mobility patterns. Detailed demographic reporting should accompany all studies to enhance transparency and enable meaningful comparisons across research efforts. Future analytical approaches should incorporate algorithms capable of capturing temporal patterns to model fluctuations in stress over time. Rather than relying solely on binary stress categorizations, researchers should develop models that characterize stress as a dynamic and progressive state, allowing for the detection of mild, moderate, and chronic stress levels. Clear explanations of baseline stress measurements are also needed to ensure that resting conditions are consistently defined and comparable across studies. Finally, stress prediction models should increasingly focus on personalization while maintaining robust privacy protections for participants.

## Supplementary material

10.2196/64144 Multimedia Appendix 1 Search terms and phrases.

10.2196/64144 Multimedia Appendix 2 Quality assessment scoring details.

10.2196/64144 Multimedia Appendix 3 Quality scores by paper.

10.2196/64144 Multimedia Appendix 4 Study key and publication information.

10.2196/64144 Checklist 1 PRISMA-ScR checklist.

## Abbreviations

CNN: convolutional neural network
ECG: electrocardiogram
EDA: electrodermal activity
HR: heart rate
HRV: heart rate variability
LSTM: long short-term memory
PRISMA-S: Preferred Reporting Items for Systematic Reviews and Meta-Analyses literature search extension
PRISMA-ScR: Preferred Reporting Items for Systematic Reviews and Meta-Analyses Extension for Scoping Reviews
RF: random forest
SVM: support vector machine
TSST: Trier Social Stress Test
WESAD: Wearable Stress and Affect Detection
XGBoost: extreme gradient boosting
