# Supplementary material for: Examining the Use of Consumer Wearable Devices and Digital Tools for Stress Measurement in College Students: Scoping Review of Methods
Source: JMIR Mhealth Uhealth. 2026 Mar 30;14:e64144. doi: 10.2196/64144 (PMC13035038; doi:10.2196/64144)
Supplement: Multimedia Appendix 1 [file mhealth-v14-e64144-s001.docx]

Pubmed: 124

(stress* OR "Stress, Psychological"[MeSH] OR “Stress, Physiological”[MeSH]) AND (((device* OR tool* OR tech* OR sensor*) AND (smart OR wearable OR “physiological monitor*”)) OR “Wearable electronic devices”[MeSH])

Embase: 152

(stress* OR 'Stress, Psychological'/exp OR 'Stress, Physiological'/exp) AND (((device* OR tool* OR tech* OR sensor*) AND (smart OR wearable OR 'physiological monitor*')) OR 'Wearable electronic devices'/exp)

ACM Digital Library: 104

(stress* OR "psychological stress” OR “physiological stress”) AND (((device* OR tool* OR tech* OR sensor*) AND (smart OR wearable OR “physiological monitor*”)) OR “Wearable electronic devices”)

IEEE Xplore: 412

("Abstract":stress OR "Mesh_Terms":"stress, psychological" OR "Mesh_Terms":"stress, physiological") AND ((("Abstract":device* OR "Abstract":tool* OR "Abstract":tech* OR "Abstract":sensor*) AND ("Abstract":smart OR "Abstract":wearable OR "Abstract":"physiological monitor*")) OR "Abstract":"wearable electronic devices")
