# Supplementary material for: Examining the Use of Consumer Wearable Devices and Digital Tools for Stress Measurement in College Students: Scoping Review of Methods
Source: JMIR Mhealth Uhealth. 2026 Mar 30;14:e64144. doi: 10.2196/64144 (PMC13035038; doi:10.2196/64144)
Supplement: Multimedia Appendix 2 [file mhealth-v14-e64144-s002.docx]

Definition of Outcomes: 2, clear definition of outcomes. 1, unclear definition of outcomes. 0, no definition of outcomes reported.

Sample Description and Eligibility Definition: 2, well-defined eligibility criteria and methods for sample selection. 1, incomplete or unclear eligibility criteria or poorly documented methods for sample selection. 0, no sampling strategy or eligibility.

Representativeness: 2, representative sample of target population. 1, partially representative sampling with potential sampling bias. 0, demographics not mentioned or sample not representative.

Justification of Sample Size: 2, clear and appropriate justification of sample size. 1, unclear or partial justification of sample size. 0, no justification of sample size given.
