# Supplementary material for: Examining the Use of Consumer Wearable Devices and Digital Tools for Stress Measurement in College Students: Scoping Review of Methods
Source: JMIR Mhealth Uhealth. 2026 Mar 30;14:e64144. doi: 10.2196/64144 (PMC13035038; doi:10.2196/64144)
Supplement: Multimedia Appendix 3 [file mhealth-v14-e64144-s003.docx]

| **Study ID** | **Definition of Outcomes** | **Sample Description and Eligibility Definition** | **Representativeness** | **Justification of Sample Size** |  |  |  |  |  |
| --- | --- | --- | --- | --- | --- | --- | --- | --- | --- |
| 1 | 2 | 2 | 1 | 0 |  |  |  |  |  |
| 2 | 2 | 1 | 0 | 0 |  |  |  |  |  |
| 3 | 1 | 1 | 0 | 0 |  |  |  |  |  |
| 4 | 2 | 1 | 0 | 0 |  |  |  |  |  |
| 5 | 2 | 2 | 1 | 0 |  |  |  |  |  |
| 6 | 2 | 2 | 1 | 0 |  |  |  |  |  |
| 7 | 2 | 2 | 1 | 1 |  |  |  |  |  |
| 8 | 2 | 1 | 0 | 0 |  |  |  |  |  |
| 9 | 2 | 2 | 1 | 0 |  |  |  |  |  |
| 10 | 2 | 2 | 2 | 1 |  |  |  |  |  |
| 11 | 1 | 2 | 1 | 1 |  |  |  |  |  |
| 12 | 2 | 2 | 1 | 0 |  |  |  |  |  |
| 13 | 2 | 2 | 0 | 0 |  |  |  |  |  |
| 14 | 2 | 2 | 1 | 0 |  |  |  |  |  |
| 15 | 1 | 1 | 0 | 0 |  |  |  |  |  |
| 16 | 2 | 2 | 0 | 0 |  |  |  |  |  |
| 17 | 2 | 1 | 1 | 0 |  |  |  |  |  |
| 18 | 2 | 2 | 1 | 0 |  |  |  |  |  |
| 19 | 2 | 2 | 2 | 1 |  |  |  |  |  |
| 20 | 2 | 2 | 1 | 0 |  |  |  |  |  |
| 21 | 2 | 2 | 1 | 0 |  |  |  |  |  |
| 22 | 2 | 1 | 0 | 0 |  |  |  |  |  |
| 23 | 2 | 2 | 1 | 0 |  |  |  |  |  |
| 24 | 2 | 2 | 1 | 0 |  |  |  |  |  |
| 25 | 2 | 1 | 0 | 0 |  |  |  |  |  |
| 26 | 2 | 2 | 1 | 0 |  |  |  |  |  |
| 27 | 2 | 2 | 1 | 0 |  |  |  |  |  |
| 28 | 2 | 1 | 0 | 1 |  |  |  |  |  |
| 29 | 2 | 2 | 1 | 0 |  |  |  |  |  |
| 30 | 2 | 2 | 1 | 0 |  |  |  |  |  |
| 31 | 1 | 1 | 0 | 0 |  |  |  |  |  |
| 32 | 2 | 2 | 1 | **2** |  |  |  |  |  |
| 33 | 2 | 2 | 1 | 0 |  |  |  |  |  |
| 34 | 2 | 2 | 1 | 0 |  |  |  |  |  |
| 35 | 2 | 2 | 1 | 0 |  |  |  |  |  |
| 36 | 1 | 2 | 1 | 0 |  |  |  |  |  |
| 37 | 2 | 2 | 1 | 0 |  |  |  |  |  |
| 38 | 2 | 2 | 1 | 0 |  |  |  |  |  |
| 39 | 2 | 2 | 2 | 1 |  |  |  |  |  |
| 40 | 2 | 2 | 2 | 0 |  |  |  |  |  |
| 41 | 2 | 2 | 1 | 1 |  |  |  |  |  |
| 42 | 2 | 2 | 1 | 0 |  |  |  |  |  |
| 43 | 2 | 1 | 1 | 0 |  |  |  |  |  |
| 44 | 2 | 1 | 1 | 0 |  |  |  |  |  |
| 45 | 2 | 2 | 1 | 0 |  |  |  |  |  |
| 46 | 2 | 1 | 1 | 0 |  |  |  |  |  |
| 47 | 2 | 2 | 1 | 0 |  |  |  |  |  |
| 48 | 2 | 1 | 1 | 0 |  |  |  |  |  |
| 49 | 2 | 1 | 1 | 0 |  |  |  |  |  |
| 50 | 2 | 1 | 1 | 0 |  |  |  |  |  |
| 51 | 2 | 1 | 1 | 0 |  |  |  |  |  |
| 52 | 2 | 2 | 1 | 0 |  |  |  |  |  |
| 53 | 2 | 1 | 1 | 0 |  |  |  |  |  |
| 54 | 2 | 1 | 1 | 0 |  |  |  |  |  |
| 55 | 2 | 1 | 1 | 0 |  |  |  |  |  |
| 56 | 2 | 1 | 1 | 0 |  |  |  |  |  |
| 57 | 2 | 1 | 1 | 0 |  |  |  |  |  |
| 58 | 2 | 1 | 1 | 0 |  |  |  |  |  |
| 59 | 2 | 1 | 1 | 0 |  |  |  |  |  |
| 60 | 2 | 1 | 1 | 0 |  |  |  |  |  |
| 61 | 2 | 1 | 1 | 0 |  |  |  |  |  |
| 62 | 2 | 2 | 1 | 0 |  |  |  |  |  |
| 63 | 2 | 1 | 1 | 0 |  |  |  |  |  |
| 64 | 2 | 1 | 1 | 0 |  |  |  |  |  |
| 65 | 2 | 1 | 1 | 0 |  |  |  |  |  |
| 66 | 2 | 2 | 1 | 0 |  |  |  |  |  |
| 67 | 2 | 1 | 1 | 0 |  |  |  |  |  |
| 68 | 2 | 1 | 1 | 0 |  |  |  |  |  |
| 69 | 2 | 1 | 1 | 0 |  |  |  |  |  |
| 70 | 2 | 1 | 1 | 0 |  |  |  |  |  |
| 71 | 2 | 1 | 1 | 0 |  |  |  |  |  |
| 72 | 2 | 2 | 1 | 0 |  |  |  |  |  |
| 73 | 2 | 1 | 1 | 0 |  |  |  |  |  |
| 74 | 2 | 1 | 1 | 0 |  |  |  |  |  |
| 75 | 1 | 1 | 1 | 0 |  |  |  |  |  |
| 76 | 2 | 1 | 1 | 0 |  |  |  |  |  |
| 77 | 2 | 1 | 1 | 0 |  |  |  |  |  |
| 78 | 2 | 1 | 1 | 0 |  |  |  |  |  |
| 79 | 2 | 1 | 1 | 0 |  |  |  |  |  |
| 80 | 2 | 1 | 1 | 0 |  |  |  |  |  |
| 81 | 2 | 1 | 1 | 0 |  |  |  |  |  |
| 82 | 2 | 1 | 1 | 0 |  |  |  |  |  |
| 83 | 2 | 2 | 1 | 0 |  |  |  |  |  |
| 84 | 2 | 2 | 1 | 0 |  |  |  |  |  |
| 85 | 2 | 1 | 0 | 0 |  |  |  |  |  |
| 86 | 2 | 1 | 1 | 0 |  |  |  |  |  |
| 87 | 2 | 1 | 0 | 0 |  |  |  |  |  |
| 88 | 2 | 1 | 1 | 0 |  |  |  |  |  |
| 89 | 2 | 0 | 0 | 0 |  |  |  |  |  |
| 90 | 2 | 1 | 1 | 0 |  |  |  |  |  |
| 91 | 2 | 1 | 1 | 0 |  |  |  |  |  |
| 92 | 2 | 1 | 1 | 1 |  |  |  |  |  |
| 93 | 2 | 1 | 1 | 0 |  |  |  |  |  |
| 94 | 2 | 1 | 1 | 0 |  |  |  |  |  |
| 95 | 2 | 1 | 1 | 0 |  |  |  |  |  |
| 96 | 2 | 1 | 1 | 0 |  |  |  |  |  |
| 97 | 2 | 0 | 0 | 0 |  |  |  |  |  |
| 98 | 2 | 1 | 1 | 0 |  |  |  |  |  |
| 99 | 2 | 1 | 1 | 0 |  |  |  |  |  |
| 100 | 2 | 2 | 1 | 0 |  |  |  |  |  |
| 101 | 2 | 1 | 1 | 0 |  |  |  |  |  |
| 102 | 2 | 1 | 1 | 0 |  |  |  |  |  |
| 103 | 2 | 0 | 0 | 0 |  |  |  |  |  |
| 104 | 2 | 1 | 0 | 0 |  |  |  |  |  |
| 105 | 2 | 1 | 1 | 0 |  |  |  |  |  |
| 106 | 2 | 1 | 1 | 0 |  |  |  |  |  |
| 107 | 2 | 1 | 1 | 0 |  |  |  |  |  |
| 108 | 2 | 1 | 1 | 0 |  |  |  |  |  |
| 109 | 1 | 1 | 1 | 0 |  |  |  |  |  |
| 110 | 2 | 1 | 1 | 0 |  |  |  |  |  |
| 111 | 2 | 0 | 0 | 0 |  |  |  |  |  |
| 112 | 2 | 0 | 0 | 0 |  |  |  |  |  |
| 113 | 2 | 1 | 1 | 0 |  |  |  |  |  |
| 114 | 2 | 0 | 0 | 0 |  |  |  |  |  |
| 115 | 2 | 0 | 0 | 0 |  |  |  |  |  |
| 116 | 2 | 1 | 1 | 0 |  |  |  |  |  |
| 117 | 2 | 1 | 1 | 0 |  |  |  |  |  |
| 118 | 2 | 2 | 1 | **2** |  |  |  |  |  |
| 119 | 2 | 1 | 1 | 0 |  |  |  |  |  |
| 120 | 2 | 1 | 1 | 0 |  |  |  |  |  |
| 121 | 2 | 0 | 0 | 0 |  |  |  |  |  |
| 122 | 2 | 0 | 0 | 0 |  |  |  |  |  |
| 123 | 2 | 2 | 1 | 1 |  |  |  |  |  |
| 124 | 2 | 1 | 1 | 0 |  |  |  |  |  |
| 125 | 2 | 1 | 1 | 0 |  |  |  |  |  |
| 126 | 2 | 1 | 1 | 0 |  |  |  |  |  |
| 127 | 2 | 1 | 1 | 0 |  |  |  |  |  |
| 128 | 2 | 1 | 1 | 0 |  |  |  |  |  |
| 129 | 2 | 1 | 1 | 0 |  |  |  |  |  |
| 130 | 2 | 1 | 1 | 0 |  |  |  |  |  |
| 131 | 2 | 1 | 1 | 0 |  |  |  |  |  |
| 132 | 2 | 1 | 1 | 0 |  |  |  |  |  |
| 133 | 2 | 1 | 1 | **2** |  |  |  |  |  |
| 134 | 2 | 1 | 1 | **2** |  |  |  |  |  |
