# Supplementary material for: Examining the Use of Consumer Wearable Devices and Digital Tools for Stress Measurement in College Students: Scoping Review of Methods
Source: JMIR Mhealth Uhealth. 2026 Mar 30;14:e64144. doi: 10.2196/64144 (PMC13035038; doi:10.2196/64144)
Supplement: Multimedia Appendix 4 [file mhealth-v14-e64144-s004.docx]

| **Paper** | **Study ID** | **Author(s)** | **Publication Year** |
| --- | --- | --- | --- |
| EMoCy: Towards Physiological Signals-Based Stress Detection[38] | 1 | Armando Bellante, Letizia Bergamasco, Ana Bogdanovic, Noemi Gozzi, Lorenzo Gecchelin, Moaad Khamlich, Anisia Lauditi, Eleonora D’Arnese, Marco D. Santambrogio | 2021 |
| Prognostics and Management of Mental Stress By AIoT Monitoring and Schlegel Diagrams[39] | 2 | Alberto Faro and Daniela Giordano | 2021 |
| Finding the Proper Mental Stress Model Depending on Context using Edge Devices and Machine Learning[40] | 3 | Alberto Faro, Daniela Giordano, Mario Venticinque | 2020 |
| Relearn: A Robust Machine Learning Framework in Presence of Missing Data for Multimodal Stress Detection from Physiological Signs[41] | 4 | Arman Iranfar, Afriana Arza, David Atienza | 2021 |
| An Integrated Human Stress Detection Sensor Using Supervised Algorithms[42] | 5 | Amirmohammad Mohammadi, Mohammad Fakharzadeg, Senior Member, IEEE, and Bardia Baraeinejad | 2022 |
| Stress Detector System Using IoT and Artificial Intelligence[43] | 6 | Areej Mustafa, Maitha Alahmed, Aysha Alhammadi | 2020 |
| Human Stress Classification During Public Speaking Using Physiological Signals[44] | 7 | Aamir Arsalan, Muhammad Majid | 2021 |
| Early versus Late Modality Fusion of Deep Wearable Sensor Features for Personalized Prediction of Tomorrow’s Mood, Health, Stress[45] | 8 | Boning Li and Akane Sano | 2020 |
| How Laboratory Experiments Can Be Exploited For Monitoring Stress in the Wild: A Bridge Between Laboratory and Daily Life[27] | 9 | Yekta Said Can, Dilara Gokay, Dilruba Reyyan Kilic, Deniz Ekiz, Niaz Chalabianloo, Cen Esroy | 2020 |
| Race and ethnic variation in college students’ allostatic regulation of racism-related stress[46] | 10 | Jacob E. Cheadle, Bridget J. Goosby, Joseph C. Jochman, Cara C. Tomaso, Chelsea B. Kozikowski Yancey, Timothy D. Nelson | 2020 |
| A multi-feature and Time-aware-based Stress Evaluation Mechanism for Mental Status Adjustment[47] | 11 | Min Chen, Wenjing Xiao, Miao Li, Yixue Hao, Long hu, Guangming Tao | 2022 |
| Resolving Data Overload and Latency Issues in Multivariate Time-Serie IoMT Data for Mental Health Monitoring[48] | 12 | Divya Gupta, M.P.S. Bhatia, Akshi Kumar | 2021 |
| Stress Detection Using Smartphone Extracted Photoplethysmography[49] | 13 | Francis C. Panganiban, Franz A. de Leon | 2021 |
| A Deep Learning Approach to Recognize Cognitive Load using PPG Signals[50] | 14 | Francesca Gasparini, Alessandra Grossi, Stefania Bandini | 2021 |
| Closed-Loop Cognitive Stress Regulation Using Fuzzy Control in Wearable-machine interface Architectures[51] | 15 | Hamid Fekri Azgomi, Iahn Cajigas, Rose T. Faghih | 2021 |
| Passive Sensor Data Based Future Mood, Health, and Stress Prediction: User Adaptation Using Deep Learning[31] | 16 | Han Yu, Akane Sano | 2020 |
| Objective Stress Monitoring Based on Wearable Sensors in Everyday Settings[52] | 17 | Hee Jeong Han | 2020 |
| Stress Detection Using Wearable Devices Based on Transfer Learning[53] | 18 | Jinting Wu, Yujia Zhang, Xiaoguang Zhao | 2021 |
| Do Trait Psychological Characteristics Moderate Sympathetic Arousal to Racial Discrimination Exposure in a Natural Setting[54] | 19 | Elizabeth B. Jelsma, Bridget J. Goosby, Jacob E. Cheadle | 2021 |
| Intelligent Stress Monitoring Assistant for First Responders[55] | 20 | Kenneth Lai, Svetlana N. Yanushkevich, Vlad P. Shmerko | 2021 |
| CNN based stress and emotion Recognition in Ambulatory Settings[56] | 21 | Leonidas Liakopoulos, Nikolaos Stagakis, Evangelia I. Zacharaki, Konstantinos Moustakas | 2021 |
| Extraction and Interpretation of Deep Autoencoder-based Temporal Features from Wearables for Forecasting Personalized Mood, Health, and Stress[57] | 22 | Boning Li, Akane Sano | 2020 |
| Multi-Modal Physiological Data Fusion for Affect Estimation Using Deep Learning[58] | 23 | Murtadha D. Hssayeni, Behnaz Ghoraani | 2021 |
| Human Stress Detection with Wearable Sensors Using Convolutional Neural Networks[59] | 24 | Manuel Gil-Martin, Ruben San-Segundo, Ana Mateos, Javier Ferreiros-Lopez | 2022 |
| Disentangled Adversarial Transfer Learning for Physiological Biosignals[60] | 25 | Mo Han, Ozan Ozdenizci, Ye Wang, Toshiaki Koike-Akino, Deniz Erdogmus | 2020 |
| Continuous Detection of Physiological Stress with Commodity Hardware[61] | 26 | Varun Mishra, Gunnar Pope, Sarah Lord, Stephanie Lewia, Byron Lowens, Kelly Cane, Sougata Sen, Ryan Halter, David Kotz | 2020 |
| Evaluating the Reproducibility of Physiological Stress Detection Models[26] | 27 | Varun Mishra, Sougata Sen, Grace Chen, Tian Hao, Jeffrey Rogers, Ching-Hua Chen, David Kotz | 2020 |
| CAFS: Cost-aware Features Selection Method for Multimodal Stress Monitoring on Wearable Devices[62] | 28 | Niloofar Momeni, Adriana Arza Valdes, Joao Rodrigues, Carmen Sandi, David Atienza | 2021 |
| Feature Augmented Hybrid CNN for Stress Recognition Using Wrist-based Photoplethysmography Sensor[63] | 29 | Nafiul Rashid, Luke Chen, Manik Dautta, Abel Jimenez, Peter Tseng, Mohammad Abdullah Al Faruque | 2021 |
| Stress Detection With Machine Learning and Deep Learning using Multimodal Physiological Data[18] | 30 | Pramod Bobade, Vani M | 2020 |
| Research Study and System Design For Evaluating Student Stress in Indian Academic Setting[64] | 31 | Pavan Kumar Reddy Yannam, Vineet Venkatesh, Manik Gupta | 2022 |
| Using Consumer-wearable Technology for Remote Assessment of Physiological Response to Stress in the Naturalistic Environment[65] | 32 | Serguei V. S. Pakhomov, Paul D. Thuras, Raymond Finzel, Jerika Eppel, Michael Kotlyar | 2020 |
| Comparing the Predictability of Sensor Modalities to Detect Stress From Wearable Sensor Data[66] | 33 | Ryan Holder, Ramesh Kumar Sah, Michael Cleveland, Hassan Ghasemzadeh | 2022 |
| Automatic and Intelligent Stressor Identification Based on Photoplethysmography Analysis[67] | 34 | Sam Elzeiny, Marwa Qaraqe | 2021 |
| Stress Detection With Single PPG Sensor by Orchestrating Multiple Denoising and Peak-Detecting Methods[68] | 35 | Seongsil Heo, Sunyoung Kwon, Jaekoo Lee | 2021 |
| Assessment of Mental Stress From Limited Features Based on GRU-RNN[69] | 36 | Sambit Prasad Kar, Nirmal Kumar Rout, Jonothan Joshi | 2021 |
| Digital Machine Learning Circuit for Real-Time Stress Detection from Wearable ECG Sensor[70] | 37 | Samukh Prashant Bhanushali, Sudarsan Sadasivuni, Imin Banerjee, Arindam Sanyal | 2020 |
| Stress Detection Via Sensor Translation[71] | 38 | Sirat Samyoun, Abu Sayeed Mondol, John A. Stankovic | 2020 |
| Stress Among Portuguese Medical Students: the EuStress Solution[72] | 39 | Eliana Silva, Joyce Aguiar, Luis Paulo Reis, Jorge Oliveira e Sa, Joaquim Goncalves, Victor Carvalho | 2020 |
| College Life is Hard! Shedding Light on Stress Prediction for Autistic College Students using Data-Driven Analysis[73] | 40 | Tanzima Z. Islam, Philip Wu Liang, Forest Sweeney, Cody Pragner, Jayaraman J. Thiagarajan, Moushimi Sharmin, Shameem Ahmed | 2021 |
| Fluctuations in Behavior and Affect in College Students Measured Using Deep Phenotyping[32] | 41 | Constanza M. Vidal Bustamante, Garth Coombs, Habiballah Rahimi-Eichi, Patrick Mair, Jukka-Pekka Onnela, Justin T. Baker, Randy L. Buckner | 2022 |
| Unsupervised Learning Method for Exploring Students’ Mental Stress in Medical Simulation Training[74] | 42 | Yujin WU, Mohammed Daoudi, Ali Amad, Laurent Sparrow, Fabien D’Hondt | 2020 |
| AI-Enabled Smart Wristband Providing Real-Time Vital Signs and Stress Monitoring[75] | 43 | Nikos Mitro, Katerina Argyri, Lampros Pavlopoulos, Dimitrios Kosyvas, Lazaros Karagiannidis, Margarita Kostovasili, Fay Misichroni, Eleftherios Ouzounoglou, Angelos Amditis | 2023 |
| Stress Detection Through Wrist-Based Electrodermal Activity Monitoring and Machine Learning[28] | 44 | Lili Zhu, Petros Spachos, Pai Chet Ng, Yuanhao Yu, Yang Wang, Konstantinos Plataniotis, Dimitrios Hatzinakos | 2023 |
| Detecting Prolonged Stress in Real Life Using Wearable Biosensors and Ecological Momentary Assessments: Naturalistic Experimental Study[76] | 45 | Rayyan Tutunji, Nikos Kogias, Bob Kapteijns, Martin Krentz, Florian Krause, Eliana Vassena, Erno Hermans. | 2023 |
| Generating Synthetic Health Sensor Data for Privacy-Preserving Wearable Stress Detection[77] | 46 | Lucas Lange, Nils Wenzlitschke, Erhard Rahm | 2024 |
| One-Channel Wearable Mental Stress State Monitoring System[78] | 47 | Lamis Abdul Kader, Fares Al-Shargie, Usman Tariq, Hasan Al-Nashash | 2024 |
| Wrist-Based Electrodermal Activity Monitoring for Stress Detection Using Federated Learning[79] | 48 | Ahmad Almadhor, Gabriel Avelino Sampedro, Mideth Abisado, Sidra Abbas, Ye-Jin Kim, Muhammad Attique Khan, Jamel Baili, Jae-Hyuk Cha | 2023 |
| Ensemble machine learning model trained on a new synthesized dataset generalizes well for stress prediction using wearable devices[29] | 49 | Gideon Vos, Kelly Trinh, Zoltan Sarnyai, Mostafa Rahimi Azghadi | 2023 |
| On-Chip Mental Stress Detection: Integrating a Wearable Behind-The-Ear EEG Device With Embedded Tiny Neural Network[80] | 50 | Ngoc-Dau Mai, Wan-Young Chung | 2025 |
| Assessing Physiological Stress Responses in Student Nurses Using Mixed Reality Training[81] | 51 | Kamelia Sepanloo, Daniel Shevelev, Young-Jun Son, Shravan Aras, Janine E. Hinton | 2025 |
| Analyzing mental stress in Indian students through advanced machine learning and wearable technologies[2] | 52 | Shruti Gedam, Sandip Dutta, Ritesh Jha | 2025 |
| From lab to real-life: A three-stage validation of wearable technology for stress monitoring[82] | 53 | Basil A. Darwish, Shafiq Ul Rehman, Ibrahim Sadek, Nancy M. Salem, Ghada Kareem, Lamees N. Mahmoud | 2025 |
| Application of DIY Electrodermal Activity Wristband in Detecting Stress and Affective Responses of Students[83] | 54 | Kenneth Y. T. Lim, Minh Tuan Nguyen Thien, Minh Anh Nguyen Duc, Hugo F. Posada-Quintero | 2024 |
| Predicting stress in first-year college students using sleep data from wearable devices[3] | 55 | Laura S. P. Bloomfield, Mikaela I. Fudolig, Julia Kim, Jordan Llorin, Juniper L. Lovato, Ellen W. McGinnis, Ryan S. McGinnis, Matt Price, Taylor H. Ricketts, Peter Sheridan Dodds, Kathryn Stanton, Christopher M. Danforth | 2024 |
| Improved method for stress detection using bio-sensor technology and machine learning algorithms[84] | 56 | Mohd Nazeer, Shailaja Salagrama, Pardeep Kumar, Kanhaiya Sharma, Deepak Parashar, Mohammed Qayyum, Gouri Patil | 2024 |
| Efficient Feature-Selection-Based Stacking Model for Stress Detection Based on Chest Electrodermal Activity[85] | 57 | Ahmad Almadhor, Gabriel Avelino Sampedro, Mideth Abisado, Sidra Abbas | 2023 |
| Stress Detection Using Frequency Spectrum Analysis of Wrist-Measured Electrodermal Activity[86] | 58 | Žiga Stržinar, Araceli Sanchis, Agapito Ledezma, Oscar Sipele, Boštjan Pregelj, Igor Škrjanc | 2023 |
| Deep Learning Models for Stress Analysis in University Students: A Sudoku-Based Study[30] | 59 | Qicheng Chen and Boon Giin Lee | 2023 |
| Affect and stress detection based on feature fusion of LSTM and 1DCNN[87] | 60 | Mingxu Feng, Tianshu Fang, Chaozhu He, Mengqian Li & Jizhong Liu | 2024 |
| BrainNet: an automated approach for brain stress prediction utilizing electrodermal activity signal with XLNet model[88] | 61 | Liao Xuanzhi, Abeer Hakeem, Linda Mohaisen, Muhammad Umer, Muhammad Attique Khan, Shrooq Alsenan, Shtwai Alsubai, Nisreen Innab | 2024 |
| Precision Assessment of Real-World Associations Between Stress and Sleep Duration Using Actigraphy Data Collected Continuously for an Academic Year: Individual-Level Modeling Study[89] | 62 | Constanza M Vidal Bustamante, Garth Coombs III, Habiballah Rahimi-Eichi, Patrick Mair, Jukka-Pekka Onnela, Justin T Baker, Randy L Buckner | 2024 |
| Improving Stress Detection Using Weighted Score-Level Fusion of Multiple Sensor[90] | 63 | Muhammad Ali Fauzi, Bian Yang, Prosper Yeng | 2022 |
| Active Reinforcement Learning for Personalized Stress Monitoring in Everyday Settings[91] | 64 | Ali Tazarv, Sina Labbaf, Amir Rahmani, Nikil Dutt, Marco Levorato | 2023 |
| "That Student Should be a Lion Tamer!" StressViz: Designing a Stress Analytics Dashboard for Teachers[92] | 65 | Riordan Dervin Alfredo, Lanbing Nie, Paul Kennedy, Tamara Power, Carolyn Hayes, Hui Chen, Carolyn McGregor, Zachari Swiecki, Dragan Gašević, Roberto Martinez-Maldonado | 2023 |
| Random Forest Model Predicts Stress Level in a Sample of 18,403 College Students[93] | 66 | Yuzhu Su, Likun Ge, Gaoxia Wei | 2024 |
| ECG Stress Detection Model Based on Heart Rate Variability Feature Extraction[94] | 67 | Ling Wang, Jiayu Hao, Tie Hua Zhou, Fangjie Song | 2023 |
| Performance Exploration of RNN Variants for Recognizing Daily Life Stress Levels by Using Multimodal Physiological Signals[95] | 68 | Yekta Said Can, Elisabeth André | 2023 |
| Stressor Type Matters! --- Exploring Factors Influencing Cross-Dataset Generalizability of Physiological Stress Detection[96] | 69 | Pooja Prajod, Bhargavi Mahesh, Elisabeth André | 2024 |
| TinyML Based Stress Detection utilizing PPG Signals: A Lightweight Approach for Smart Wearable Devices[97] | 70 | Priyanka Ganesan, Yogeswar Reddy Thota, Hashem Shehata, Tooraj Nikoubin | 2025 |
| Stress recognition based on the Markov Transition Field of Electrodermal Activity[98] | 71 | Xue Sun, Lanjun Zhao, Rui Gao, Xinpei Wang | 2025 |
| Using Wearables to Unobtrusively Identify Periods of Stress in a Real University Environment[99] | 72 | Peter Neigel, Andrew Vargo, Benjamin Tag, Koichi Kise | 2024 |
| Multi-task Learning for Stress Recognition[100] | 73 | Alessandro Pogliaghi, Elena Di Lascio, Shkurta Gashi, Emanuela Piciucco, Silvia Santini, Martin Gjoreski | 2022 |
| GSR Based Generic Stress Prediction System[101] | 74 | Dibyanshu Jaiswal, Debatri Chatterjee, Mithun B S, Ramesh Kumar Ramakrishnan, Arpan Pal | 2023 |
| Stress Detection Using Context-Aware Sensor Fusion From Wearable Devices[102] | 75 | Nafiul Rashid, Trier Mortlock, Mohammad Abdullah Al Faruque | 2023 |
| Multi-Sensory Stress Detection System[103] | 76 | Nikita Narwat, Hitesh Kumar, Jitendra Singh Jadon, Archana Singh | 2024 |
| Stress Classification Utilising AI Studio[104] | 77 | Júlia Kafková, Rastislav Pirník, Aleš Janota, Pavol Kuchar | 2025 |
| Screening Students for Stress Using Fitbit Data[105] | 78 | Rebecca Lopez, Avantika Shrestha, Kevin Hickey, Xingtong Guo, ML Tlachac, Shichao Liu, Elke A. Rundensteiner | 2024 |
| Real-Time Stress Detection and Management Using IoT Sensors and Virtual Reality Technology[106] | 79 | Joshua Juninho Wilfred, Pavan B, Dr. R. Nirosha | 2025 |
| TinyStressNet: On-device Stress Assessment with Wearable Sensors on Edge Devices[107] | 80 | Dibyanshu Jaiswal, Shalini Mukhopadhyay, Varsha Sharma | 2024 |
| Development of a Polymeric Optical Fiber Sensor for Stress Estimation: A Comparative Analysis Between Physiological Sensors[108] | 81 | María Gaitán-Padilla, Marcela Múnera, Maria José Pontes, Marcelo Eduardo Vieira Segatto, Carlos A. Cifuentes, Camilo A. R. Diaz | 2024 |
| Multimodal Wearable Sensors-based Stress and Affective States Prediction Model[109] | 82 | Rohit Gupta, Amit Bhongade, Tapan Kumar Gandhi | 2023 |
| Automating the Development of Stress Detection Systems[110] | 83 | Felix Beierle, Rüdiger Pryss | 2023 |
| Performance Analysis of Ensemble and DNN Models for Decoding Mental Stress Utilizing ECG-Based Wearable Data Fusion[111] | 84 | Noor Masrur; Nabarun Halder; Sami Rashid; Jahanggir Hossain Setu; Ashraful Islam; Tarem Ahmed | 2024 |
| Psychological Stress Classification Using Extreme Gradient Boosting Algorithm[112] | 85 | Maria Mahardini Sakanti; Viacheslav Siniaev; Aurelia Amaris; Win-Jet Luo; Suhartono; C. Bambang Dwi Kuncoro | 2024 |
| Stress Detection Using Multimodal Physiological Signals With Machine Learning From Wearable Devices[113] | 86 | Pranita Subhash Shedage; Seyedamin Pouriyeh; Reza M. Parizi; Meng Han; Giovanna Sannino; Nasrin Dehbozorgi | 2024 |
| Stress Classification Using a Low-Cost Optical Fiber Physiological Sensor: A Preliminary Study[114] | 87 | María Gaitán-Padilla; Marcela Múnera; Carlos A. Cifuentes; Maxwell E. Monteiro; Maria J. Pontes; Camilo A. R. Diaz | 2023 |
| FuSeR: Fusion of wearables data for StrEss Recognition using explainable artificial intelligence models[115] | 88 | Ritu Tanwar; Ghanapriya Singh; Pankaj Kumar Pal | 2023 |
| A Framework for Extracting Heart Rate Variability Features from Earbud-PPG for Stress Detection[116] | 89 | Bhanu Teja Gullapalli; Viswam Nathan; Md Mahbubur Rahman; Jilong Kuang; Jun Alex Gao | 2024 |
| Machine Learning based Assessment of Mental Stress using Wearable Sensors[117] | 90 | Safia Sadruddin; Vaishali D. Khairnar; Deepali R. Vora | 2024 |
| High-Accuracy Stress Detection Using Wrist-Worn PPG Sensors[118] | 91 | Anice Jahanjoo; Nima TaheriNejad; Amin Aminifar | 2024 |
| Stress Beats: A Continuum of Learning Methods for Personalized Stress Detection[119] | 92 | Vasiliki Parousidou; Sofia Yfantidou; Christina Karagianni; Athena Vakali | 2023 |
| Physiological Data-Based Stress Detection: From Wrist Sensors to Cloud Computing and User Feedback Integration[120] | 93 | G. R. Karpagam; Harsha Vardhan V M; Kabilan K K; Pranav P; Prednya Ramesh; Suvan Sathyendira B | 2024 |
| Optimization of Wearable Biosensor Data for Stress Classification Using Machine Learning and Explainable AI[121] | 94 | Shikha Shikha; Divyashikha Sethia; S. Indu | 2024 |
| Wavelet-Based Analysis of Photoplethysmogram for Stress Detection Using Convolutional Neural Networks[122] | 95 | Yasin Hasanpoor; Bahram Tarvirdizadeh; Khalil Alipour; Mohammad Ghamari | 2023 |
| Stress Detection Using CNN on the WESAD Dataset[123] | 96 | D. Shirley Benita; A. Shamila Ebenezer; L Susmitha; M.S.P. Subathra; S. Jeba Priya | 2024 |
| Quantifying Exam Stress Progressions Using Electrodermal Activity and Machine Learning[124] | 97 | Abigail Hsu | 2023 |
| Investigating Functional Data Analysis for Wearable Physiological Sensor Data in Stress Evaluation[125] | 98 | Luca Carmisciano; Tobia Boschi; Francesca Chiaromonte; Franca Delmastro; Andrea Vandin | 2024 |
| Privacy-Preserved Stress Detection from Wearables using Federated Learning[126] | 99 | Lekha C Warrier; G.K Ragesh; B B Ram Samarth; Kishan Gurumurthy | 2024 |
| Temporal Dynamics of Classroom Stress: Insights from Wearable Sensors and Machine Learning[127] | 100 | Latherial Calbert; Navid Hashemi Tonekaboni | 2024 |
| Personalized Stress Detection for University Students Using Wearable Devices[1] | 101 | Thanh Hai Hoang; Tran Khanh Dang; Nguyen Thi Huyen Trang | 2025 |
| Resp-BoostNet: Mental Stress Detection From Biomarkers Measurable by Smartwatches Using Boosting Neural Network Technique[128] | 102 | Sanjay Kumar; Anshuman Raj Chauhan; Akhil; Akshi Kumar; Guang Yang | 2024 |
| Real-Time Stress Detection via Photoplethysmogram Signals: Implementation of a Combined Continuous Wavelet Transform and Convolutional Neural Network on Resource-Constrained Microcontrollers[129] | 103 | Yasin Hasanpoor; Amin Rostami; Bahram Tarvirdizadeh; Khalil Alipour; Mohammad Ghamari | 2024 |
| Machine Learning Algorithms for Stress Level Analysis Based on Skin Surface Temperature and Skin Conductance[130] | 104 | Thien Le Tran Thuan; Phuc Khang Nguyen; Quynh Nguyen Gia; Anh Tu Tran; Quoc Khai Le | 2024 |
| Contribution of EEG Signals for Students’ Stress Detection[131] | 105 | Jonah Fernandez; Raquel Martínez; Bianca Innocenti; Beatriz López | 2025 |
| Wearables Based Personalised Stress Recognition using Signal Processing and Hybrid Deep Learning Model[132] | 106 | Ritu Tanwar; Pankaj Kumar Pal; Ghanapriya Singh | 2024 |
| Study of a Hybrid CNN-SVM Model for Stress Detection with Automated Heart Rate Variability Feature Extraction Method[133] | 107 | Mengting Huang; Haidong Yang; Ningtao Sun; Guangshun Chen; Dalin Li; Tianyuan Zhu | 2024 |
| Personalized Ensemble Based Stress Detection Using Wearable Sensor Data[134] | 108 | Kyungtaek Oh; Jun Kyun Choi; Hyunseo Park; Seungjin Lee | 2025 |
| StressLLM: Large Language Models for Stress Prediction via Wearable Sensor Data[135] | 109 | Bishal Thapa; Micaela Rivas; Henry Griffith; Heena Rathore | 2025 |
| Machine and Deep Learning Models for Stress Detection Using Multimodal Physiological Data[136] | 110 | Eman Abdelfattah; Shreehar Joshi; Shreekar Tiwari | 2025 |
| Investigating Feasibility of Stress Detection from Social Media Content Through Wearables - GLOBECOM[137] | 111 | Kalliopi Tsiampa; Lili Zhu; Petros Spachos; Vassilis P. Plagianakos | 2023 |
| A Self-supervised Framework for Improved Data-Driven Monitoring of Stress via Multi-Modal Passive Sensing[138] | 112 | Shayan Fazeli, Lionel Levine, Mehrab Beikzadeh, Baharan Mirzasoleiman, Bita Zadeh, Tara Peris, Majid Sarrafzadeh | 2023 |
| Autoencoder-Based Human Stress Detection System Using Biological Signals[139] | 113 | P. Subathra; S. Malarvizhi | 2024 |
| CorLMI-FSA: An Efficient Feature Selection Approach for Stress Classification Using Physiological Signals[140] | 114 | Shikha Shikha; Divyashikha Sethia; S. Indu | 2025 |
| Optimisation of CNN Through Transferable Online Knowledge for Stress and Sentiment Classification[141] | 115 | Andreou Andreas; Constandinos X. Mavromoustakis; Houbing Song; Jordi Mongay Batalla | 2024 |
| Developing an explainable Deep Neural Network for stress detection using biosignals and human-engineered features[21] | 116 | Hyeokjong Lee, Jaewon Kim, Bohyung Han, Sang Min Park, Jooyoung Chang | 2025 |
| TranSenseFusers: A temporal CNN-Transformer neural network family for explainable PPG-based stress detection[142] | 117 | Panagiotis Kasnesis, Christos Chatzigeorgiou, Michalis Feidakis, Álvaro Gutiérrez, Charalampos Z Patrikakis | 2024 |
| Machine-learning detection of stress severity expressed on a continuous scale using acoustic, verbal, visual, and physiological data: lessons learned[143] | 118 | Marketa Ciharova , Khadicha Amarti, Ward van Breda, Martin J Gevonden, Sina Ghassemi, Annet Kleiboer, Christiaan H Vinkers, Milou S C Sep, Sophia Trofimova, Alexander C Cooper, Xianhua Peng, Mieke Schulte, Eirini Karyotaki, Pim Cuijpers, Heleen Riper | 2025 |
| Evaluating the Potential of Wearable Technology in Early Stress Detection: A Multimodal Approach[144] | 119 | Basil A. Darwish, Nancy M. Salem, Ghada Kareem, Lamees N. Mahmoud, Ibrahim Sadek | 2024 |
| Effect of recurrent task-induced acute stress on task performance, vagally mediated heart rate variability, and task-evoked pupil response[145] | 120 | Joseph Nuamah | 2024 |
| Multitask Learning for Mental Health: Depression, Anxiety, Stress (DAS) Using Wearables[19] | 121 | Berrenur Saylam, Özlem Durmaz İncel | 2024 |
| Human activity recognition for analyzing stress behavior based on Bi-LSTM[146] | 122 | Phataratah Sa-Nguannarm, Ermal Elbasani, Jeong-Dong Kim | 2023 |
| Smartphone Photoplethysmography Pulse Rate Covaries With Stress and Anxiety During a Digital Acute Social Stressor[147] | 123 | Benjamin W Nelson, Helen M K Harvie, Barbie Jain, Erik L Knight, Leslie E Roos, Ryan J Giuliano | 2023 |
| Global Stress Detection Framework Combining a Reduced Set of HRV Features and Random Forest Model[148] | 124 | Kamana Dahal, Brian Bogue-Jimenez, Ana Doblas | 2023 |
| Context-Aware Stress Monitoring using Wearable and Mobile Technologies in Everyday Settings[149] | 125 | Seyed Amir Hossein Aqajari, Sina Labbaf, Phuc Hoang Tran, Brenda Nguyen, Milad Asgari Mehrabadi, Marco Levorato, Nikil Dutt, Amir M. Rahmani | 2024 |
| Feasibility study for detection of mental stress and depression using pulse rate variability metrics via various durations[150] | 126 | Yu Jiao , Xinpei Wang, Changchun Liu , Guanzheng Du , Lanjun Zhao , Huiwen Dong , Shilong Zhao , Yuanyuan Liu | 2023 |
| Beyond the books: How sleep, school belonging, and physical activity affect the mental health of students under academic stress[33] | 127 | Liu Yuting, Rabiatul-adawiah Binti Ahmad Rashid | 2025 |
| Enhancing smart healthcare with female students’ stress and anxiety detection using  machine learning[151] | 128 | Farhad Lotfi, Amin Lotfi, Matin Lotfi, Artur Bjelica & Zorica Bogdanović | 2025 |
| Brain-inspired signal processing for detecting stress during mental arithmetic tasks[23] | 129 | Kais Belwafi, Ahmed Alsuwaidi, Sami Mejri, Ridha Djemal | 2025 |
| Continual Learning Strategies for Personalized Mental Well-being Monitoring from Mobile Sensing Data[152] | 130 | Giovanni Patanè, Amelia Sorrenti, Giovanni Bellitto, Simone Palazzo | 2025 |
| A Wearable Electronic Band for Stress  Understanding Using Machine Learning[153] | 131 | P. Subathra, S. Malarvizhi, K. Ferents Koni Jiavana, Shantanu Patil | 2025 |
| Stress Severity Detection in College Students Using Emotional Pulse Signals and Deep Learning[25] | 132 | Mi Li, Junzhe Li Yanbo Chen, Bin Hu | 2025 |
| Are you stressed or just excited? What the Garmin Stress Score can say about your mood[154] | 133 | D.J. van der Mee, Z. Koyuncu, I.L.J. Lemmers-Jansen | 2025 |
| Assessing Stress Level Scores Against Wearables-Driven Physiological Measurements[24] | 134 | Hadar Rosenbach, Alon Itzkovitch, Yori Gidron, Tom Schonberg | 2025 |
