# Supplementary material for: Examining the Use of Consumer Wearable Devices and Digital Tools for Stress Measurement in College Students: Scoping Review of Methods
Source: JMIR Mhealth Uhealth. 2026 Mar 30;14:e64144. doi: 10.2196/64144 (PMC13035038; doi:10.2196/64144)
Supplement: Checklist 1 [file mhealth-v14-e64144-s005.docx]

| **SECTION** | **ITEM** | **PRISMA-ScR CHECKLIST ITEM** | **REPORTED ON PAGE #** |
| --- | --- | --- | --- |
| **TITLE** | | | |
| Title | 1 | Examining the use of consumer wearable devices and digital tools for stress measurement in college students: a scoping review of methods | 1 |
| **ABSTRACT** | | | |
| Structured summary | 2 | Background: College-aged students face persistent academic and social stress that adversely affects their mental and physical health. Digital phenotyping with wearable devices enables real-time stress monitoring from continuous physiological signals, supporting just-in-time therapeutic interventions to improve student well-being. Despite rapid advances in wearables and analytical methods, it remains unclear which devices, physiological signals, and machine learning or deep learning approaches are most commonly used for stress detection in this population.  Objective: We aim to systematically review the literature to identify best practices and emerging trends in stress measurement using wearable technology and digital tools among college-aged students. We sought to evaluate commonalities in sensor types, datasets, and machine learning approaches used for stress detection.  Methods: A systematic search was conducted across medical and computer science databases, including Embase, PubMed, IEEE Xplore, and ACM Digital Library, for studies published between January 2020 and December 2025. Studies were included if they examined psychological stress detection using wearable or digital tools among college-aged students and were excluded if they focused on non-psychological stress, were reviews or prototypes without a defined study population, or lacked clear population information. Two reviewers independently screened studies and extracted data on used wearable sensors, physiological signals, datasets, and modeling approaches to summarize trends in stress prediction.  Results: 134 studies met the inclusion criteria and were included in the review out of our original 792 articles. Electrodermal activity was the most frequently used physiological signal, appearing in 57.5% of studies, and wrist-worn wearable devices were the predominant sensing modality. Among studies that compared algorithms, Support Vector Machines were identified as the most commonly applied and best-performing model in 33.3% of cases. 62.8% of included studies relied on pre-existing datasets, and approximately 80% of those used the WESAD dataset, which contains only 15 participants. Demographic reporting was inconsistent, as 27.6% of studies did not report sex distribution, and only four studies justified the sample size. The use of temporal modeling algorithms was limited, despite their importance for capturing the dynamic, time-varying nature of stress. This review highlights persistent gaps and underscores the need for more diverse datasets and advanced modeling approaches to improve stress detection accuracy and support earlier interventions for students.  Conclusions: This review provides a focused synthesis of wearable-based stress detection in college-aged students, identifying key barriers to real-world deployment, summarizing commonly used sensors, datasets, and analytical approaches. By highlighting these gaps, the review offers future guidance for developing more robust, generalizable, and temporally sensitive stress detection systems to better support early intervention and student well-being.  Keywords: digital phenotyping; wearable technology; stress detection; machine learning; college students | 1 to 2 |
| **INTRODUCTION** | | | |
| Rationale | 3 | With the widespread adoption of wearable devices, numerous stress monitoring frameworks have been designed specifically for undergraduate students, given their heightened susceptibility to psychological stress. Prior reviews have categorized stress detection approaches based on different wearable sensor types and environments, such as driving, studying, and working, assessed bio signal responses to psychological stress to evaluate their reliability and consistency, focused on model generalization when training on public datasets, or focused on broader mental-health concerns, including depression, anxiety, and stress. However, none of these reviews mapped the recent evidence on stress detection among college-aged students, a population with unique academic and social stressors, with the growing adoption of wearable technologies. Our review aims to identify trends in current stress detection research and highlight areas for improvement that future researchers should focus on. There is a need to understand which algorithms perform best, which wearables are most used, and which signals are primarily informative in stress prediction. Scoping reviews are particularly suited for these questions, where the purpose is to understand how research has been conducted, what types of evidence exist, and where inconsistencies or gaps emerge.  Being consistent with scoping-review methodology, our goal is to:   - examine how stress research is conducted in college students using consumer wearable devices and digital tools for stress measurement - identify the types of evidence available on which algorithms perform best, which wearables are most used, and which signals are most informative - clarify key concepts and methodological inconsistencies from heavy reliance on frequently used pre-existing datasets, low demographics reporting, and lack of applying temporal modeling, which can limit generalizability and real-world application   We are interested in identifying moments of high stress using digital tools/ ubiquitous data in college-aged students. We are looking at both machine learning and deep learning advancements in the field, as well as comparisons of methods, where a **scoping review** is the most appropriate synthesis method for the objectives we set out to examine. | 2 to 3 |
| Objectives | 4 | The objective of this scoping review is to map current evidence on stress detection in college-aged students using wearable and digital technologies across in-lab and daily-life contexts. Specifically, the review examines how stress is measured using physiological signals and machine-learning or deep learning methods, which sensors and datasets are most frequently used, which algorithms perform best, and what methodological gaps, exist, such as reliance on small datasets or limited demographic reporting, lack of applying temporal modeling, which can limit generalizability and real-world application. | 2 to 3 |
| **METHODS** | | | |
| Protocol and registration | 5 | There is no registration of the review protocol for our scoping review. Two independent reviewers screened all records using a two-stage selection process. Studies were checked for eligibility by them independently screening titles and abstracts. This first round of filtering focused on relevance. Abstracts were also screened for population. Some papers did not mention population in the abstract and were thus moved to full-text screening. Disagreements were resolved through discussion, and 134 studies were included. | 3 to 5 |
| Eligibility criteria | 6 | We defined eligibility criteria to ensure that only relevant and methodologically appropriate studies were included in this review. Studies were included if they measured or classified psychological stress using physiological signals from a tool, wearable, or sensor. Only experimental or observational studies published in English were considered. The target population was college students aged 18–24 years. Studies that partially included this age range were eligible if they explicitly mentioned students as a distinct group or if the mean age range, along with the standard deviation, fell within the target population.  Studies were excluded if they focused on non-psychological stress (e.g., mechanical stress), were review articles, extended abstracts, or prototype descriptions without a defined study population. Papers without clear population details or those identifying participants solely by employment (e.g., "office workers", or “hospitalized patients”) were also excluded. We were narrowing our focus to college students as the university is a particularly stressful place where their health and lifestyle habits are likely to fluctuate. | 2 to 6 |
| Information sources* | 7 | We searched IEEE Xplore, ACM Digital Library, PubMed, and Embase for conference and journal papers published between January 2020 until December 2025. We selected this publication range to receive relevant research results. We used a combination of terms related to the key concepts of psychological stress, wearables, devices, and sensors (full search per database in Multimedia Appendix 1). We confirmed that the search strategy ensured transparency and reproducibility of all search components. | 4 to 5 |
| Search | 8 | Search terms and phrases:  Pubmed: 124  *(stress* OR "Stress, Psychological"[MeSH] OR “Stress, Physiological”[MeSH]) AND (((device* OR tool* OR tech* OR sensor*) AND (smart OR wearable OR “physiological monitor*”)) OR “Wearable electronic devices”[MeSH])*  Embase: 152  *(stress* OR 'Stress, Psychological'/exp OR 'Stress, Physiological'/exp) AND (((device* OR tool* OR tech* OR sensor*) AND (smart OR wearable OR 'physiological monitor*')) OR 'Wearable electronic devices'/exp)*  ACM Digital Library: 104  *(stress* OR “psychological stress” OR “physiological stress”) AND (((device* OR tool* OR tech* OR sensor*) AND (smart OR wearable OR “physiological monitor*”)) OR “Wearable electronic devices”)*  IEEE Xplore: 412  *("Abstract":stress OR "Mesh_Terms":"stress, psychological" OR "Mesh_Terms":"stress, physiological") AND ((("Abstract":device* OR "Abstract":tool* OR "Abstract":tech* OR "Abstract":sensor*) AND ("Abstract":smart OR "Abstract":wearable OR "Abstract":"physiological monitor*")) OR "Abstract":"wearable electronic devices")*  We woud like to thank librarian Alissa Cilfone, and Lauri Fennell at Northeastern University, for their consultation regarding database search strategies and developing search terms. | 42 to 43 |
| Selection of sources of evidence† | 9 | In our scoping review, two independent reviewers screened all records using a two-stage selection process. Studies were checked for eligibility by them independently screening titles and abstracts. This first round of filtering focused on relevance. Abstracts were also screened for population. Some papers did not mention population in the abstract and were thus moved to full text screening. During this second round of filtering, studies were also checked for eligibility by two researchers independently reviewing the entire text. Disagreements at any stage of eligibility and filtering were resolved by the two researchers discussing their reasons for either inclusion, exclusion, or neither. An agreement of 100% was reached for abstract and full text screening, leading to the final inclusion of 134 papers. | 5 to 6 |
| Data charting process‡ | 10 | A data-charting form was jointly developed by two reviewers to determine which variables to extract. The reviewers independently charted the data, compared their entries, and resolved any discrepancies through discussion. The charting form was refined iteratively throughout the process, and all information was extracted directly from the published studies. | 5 to 6 |
| Data items | 11 | We extracted study characteristics, participant demographics, types of passive sensing data collected, wearable devices and physiological signals used, analytical methods, performance metrics, validation approaches, and best-performing models. Missing information was recorded as reported, and no assumptions were made about unreported variables. | 6 to 36 |
| Critical appraisal of individual sources of evidence§ | 12 | Although critical appraisal is not required for scoping reviews, we conducted an assessment of study quality to better contextualize the strengths and limitations of the included evidence. Each paper was evaluated across four categories using a three-point scoring system (0–2), as described in Multimedia Appendix 2 and reported in Multimedia Appendix 4. Following the approach of De Angelis et al., we integrated the AXIS appraisal tool for cross-sectional studies and the Newcastle–Ottawa Scale (NOS) for longitudinal studies. Studies received two points for fully meeting a criterion, one point for partial fulfillment, and zero points for non-fulfillment. These scores were used only to provide contextual interpretation and were not used to determine study inclusion or exclusion. | 31 to 60 |
| Synthesis of results | 13 | Extracted data were summarized descriptively using tables for study characteristics, algorithm comparisons, proposed frameworks, or statistical analysis. Also, visual diagrams were used to display commonly used signals, sensors, and best-performing algorithms, and study quality scores. | 6 to 60 |
| **RESULTS** | | | |
| Selection of sources of evidence | 14 | 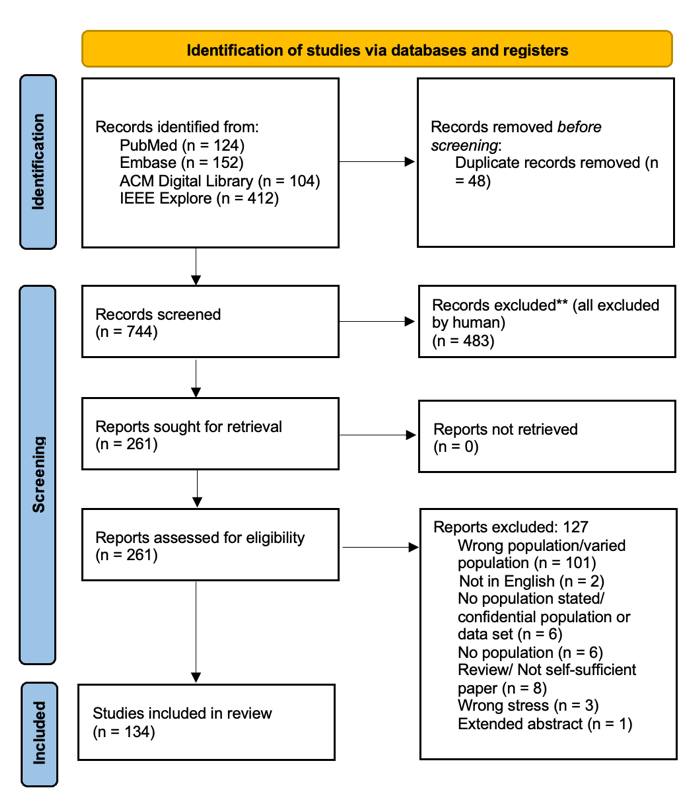 | 5 |
| Characteristics of sources of evidence | 15 | Study characteristics and extracted variables for each included article are summarized in Tables 1–3 with corresponding citations. | 6 to 31 |
| Critical appraisal within sources of evidence | 16 | Although critical appraisal is not required for scoping reviews, we conducted an assessment of study quality to better contextualize the strengths and limitations of the included evidence. Each paper was evaluated across four categories using a three-point scoring system (0–2), as described in Multimedia Appendix 2 and reported in Multimedia Appendix 4. Following the approach of De Angelis et al., we integrated the AXIS appraisal tool for cross-sectional studies and the Newcastle–Ottawa Scale (NOS) for longitudinal studies. Studies received two points for fully meeting a criterion, one point for partial fulfillment, and zero points for non-fulfillment. | 31 to 60 |
| Results of individual sources of evidence | 17 | Individual study data are summarized in manuscript tables I to III and expanded in the Multimedia Appendix, with key patterns (such as common sensor use, top physiological signals used, algorithm performance, and study quality scores) illustrated through visual diagrams,  including the PRISMA flow diagram showing study selection from medical and computer science databases. | 5 to 60 |
| Synthesis of results | 18 | 134 studies met the inclusion criteria and were included in the review out of our original 792 articles. Electrodermal activity was the most frequently used physiological signal, appearing in 57.5% of studies, and wrist-worn wearable devices were the predominant sensing modality. Among studies that compared algorithms, Support Vector Machines were identified as the most commonly applied and best-performing model in 33.3% of cases. 62.8% of included studies relied on pre-existing datasets, and approximately 80% of those used the WESAD dataset, which contains only 15 participants. Demographic reporting was inconsistent, as 27.6% of studies did not report sex distribution, and only four studies justified the sample size. The use of temporal modeling algorithms was limited, despite their importance for capturing the dynamic, time-varying nature of stress. This review highlights persistent gaps and underscores the need for more diverse datasets and advanced modeling approaches to improve stress detection accuracy and support earlier interventions for students. | 32 to 38 |
| **DISCUSSION** | | | |
| Summary of evidence | 19 | In this scoping review, we examined how stress is measured among college-aged students using wearable technologies and machine learning methods between 2020–2025, with the goal of identifying commonly used wearables, the most informative physiological signals, and the best-performing algorithms. Across the literature, we found that SVMs among traditional machine learning models and CNNs among deep learning models were the strongest performers for stress classification. Wrist-worn devices were the predominant sensor platform, and EDA was the most frequently measured and most informative signal. However, most studies relied on small, homogeneous samples, frequently used controlled laboratory datasets such as WESAD, and commonly employed binary (stressed vs. not stressed) labeling approaches, raising concerns about representativeness and ecological validity. Our quality assessment further revealed inconsistent demographic reporting, insufficient justification of sample sizes, limited attention to social determinants of stress, and substantial variation in how psychological stress was defined, elicited, and validated across studies.  Considering recent advances in sensing modalities, datasets, and analytical approaches, this review highlights these recurring gaps and emphasizes the need for more diverse datasets and advanced modeling approaches tailored to student stress patterns. Strengthening these areas could improve the accuracy and robustness of stress detection systems, enabling earlier intervention and reducing the mental health burden in students. | 36 to 41 |
| Limitations | 20 | A common challenge in the reviewed papers was the inclusion of multiple populations or datasets within a single study. While our primary focus was on college students, some papers analyzed mixed populations or multiple datasets. However, as long as college students were included, these studies were still considered in our review. Many papers also used overlapping datasets such as the WESAD dataset, although different papers used different parts of the dataset along with different models. This may lead to some redundancy in findings. Many of the commonly used datasets, such as WESAD with only 15 subjects, had limited sample sizes, introducing potential bias and reducing the likelihood of capturing a truly representative population. Additionally, only studies published in English were included as this was the language accessible to our reviewers, which may have led to the exclusion of relevant research. | 40 |
| Conclusions | 21 | Significant advancements are being made in stress measurement using wearables, including commercially available devices, creating opportunities for real-time stress detection and improved health outcomes. However, current research highlights the need for larger and more diverse samples to enhance representativeness. Many studies rely on a limited number of existing datasets, which, despite methodological differences, may result in overlapping findings. Additionally, greater diversity in sex and ethnic demographics is essential to better understand population-level impacts. To address these gaps, studies should improve demographic reporting and provide clearer justifications for sample sizes.  There is a need for stress models that better reflect the way human stress functions. Most studies categorized stress as a binary state, stressed vs. not stressed, overlooking variations in intensity, such as mild or moderate stress, which can be chronic and have significant health implications. Incorporating these nuances could improve stress detection and intervention strategies. Additionally, few studies employed algorithms like RNNs, which can capture temporal patterns, despite the importance of tracking stress progression over time. Greater emphasis on time-dependent modeling could enhance the understanding of how stress evolves. Lastly, many studies failed to clearly distinguish between psychological stress and physiological stress responses, despite the critical need for distinct measurement approaches. More precise definitions and methodologies are necessary to differentiate between these two aspects of stress effectively.  To strengthen the credibility and generalizability of future research, studies should provide clear justifications for their sample sizes and, where possible, aim to recruit larger cohorts that reduce bias and improve statistical reliability. The field would also benefit from the development and use of more varied datasets, which can limit overlap across studies and reduce potential sources of bias. Increasing diversity in participant recruitment is essential; researchers should ensure representation across race, gender, socioeconomic status, and environmental contexts, as well as variation in behavioral and lifestyle factors such as sleep duration and efficiency, physical activity, phone usage, social media engagement, and mobility patterns. Detailed demographic reporting should accompany all studies to enhance transparency and enable meaningful comparisons across research efforts.  Future analytical approaches should incorporate algorithms capable of capturing temporal patterns, such as recurrent neural networks, to model fluctuations in stress over time. Rather than relying solely on binary stress categorizations, researchers should develop models that characterize stress as a dynamic and progressive state, allowing for the detection of mild, moderate, and chronic stress levels. Clear explanations of baseline stress measurements are also needed to ensure that resting conditions are consistently defined and comparable across studies. Finally, stress prediction models should increasingly focus on personalization while maintaining robust privacy protections for participants. | 40 to 41 |
| **FUNDING** | | | |
| Funding | 22 | This study represents independent research funded by Northeastern University’s Project-Based Exploration for the Advancement of Knowledge (PEAK) Experience #2: The Base Camp Award and Northeastern University’s FY23 Transforming Interdisciplinary Experiential Research (Tier) 1 Seed Grant: Assessing the Scalability and feasibility of digitally phenotyping stress. | 42 |

JBI = Joanna Briggs Institute; PRISMA-ScR = Preferred Reporting Items for Systematic reviews and Meta-Analyses extension for Scoping Reviews.

* Where *sources of evidence* (see second footnote) are compiled from, such as bibliographic databases, social media platforms, and Web sites.

† A more inclusive/heterogeneous term used to account for the different types of evidence or data sources (e.g., quantitative and/or qualitative research, expert opinion, and policy documents) that may be eligible in a scoping review as opposed to only studies. This is not to be confused with *information sources* (see first footnote).

‡ The frameworks by Arksey and O’Malley (6) and Levac and colleagues (7) and the JBI guidance (4, 5) refer to the process of data extraction in a scoping review as data charting*.*

§ The process of systematically examining research evidence to assess its validity, results, and relevance before using it to inform a decision. This term is used for items 12 and 19 instead of "risk of bias" (which is more applicable to systematic reviews of interventions) to include and acknowledge the various sources of evidence that may be used in a scoping review (e.g., quantitative and/or qualitative research, expert opinion, and policy document).

*From:* Tricco AC, Lillie E, Zarin W, O'Brien KK, Colquhoun H, Levac D, et al. PRISMA Extension for Scoping Reviews (PRISMAScR): Checklist and Explanation. Ann Intern Med. 2018;169:467–473. [doi: 10.7326/M18-0850](http://annals.org/aim/fullarticle/2700389/prisma-extension-scoping-reviews-prisma-scr-checklist-explanation).
